# Aristolane-type Sesquiterpenoids from *Nardostachys chinensis* and Revised Structure of Aristolanhydride

**DOI:** 10.1007/s13659-019-0200-7

**Published:** 2019-03-08

**Authors:** Li-Xia Wang, Xian-Jun Jiang, Xiang-Mei Li, Mei-Fen Mao, Guo-Zhu Wei, Fei Wang

**Affiliations:** 1 BioBioPha Co., Ltd., Kunming, 650201 People’s Republic of China; 2 Reference Substances Sub-center, National Engineering Research Center for Modernization of Traditional Chinese Medicine, Kunming, 650201 People’s Republic of China

**Keywords:** *Nardostachys chinensis*, Aristolane, Secoaristolane, Aristolane-chalcone hybrid, Structural revision

## Abstract

**Abstract:**

Four hitherto unknown aristolane-type sesquiterpenes, including one novel 8,9-secoaristolane, namely secoaristolenedioic acid (**1**), two aristolone derivatives, namely 1*α*,2*β*-dihydroxyaristolone (**2**), 9-epidebilon (**3**), and one rare aristolane-chalcone hybrid, namely 3′-hydroxynardoaristolone A (**4**) were isolated from the ethanol extract of the roots and rhizomes of *Nardostachys chinensis*. Their structures were elucidated on the basis of extensive spectroscopic analysis. In addition, the structure of aristolanhydride, recently isolated from the same species, was corrected by reanalysis of the published NMR data.

**Graphical Abstract:**

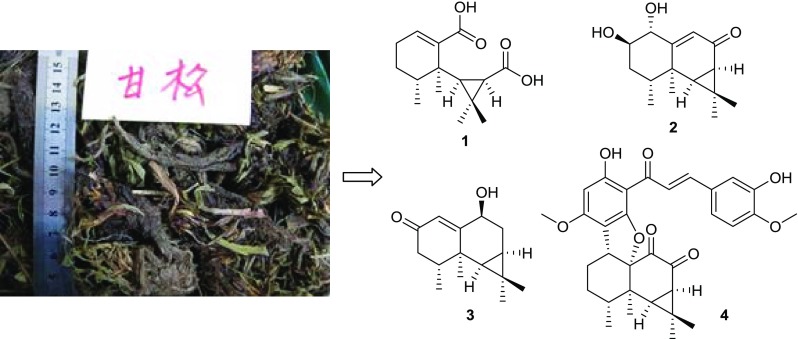

**Electronic supplementary material:**

The online version of this article (10.1007/s13659-019-0200-7) contains supplementary material, which is available to authorized users.

## Introduction

*Nardostachys chinensis* is a perennial herb belonging to the genus *Nardostachys* (Valerianaceae family), and mainly distributed in the Himalayan mountain areas [1]. The roots and rhizomes of *N. chinensis*, known as “Gansong”, is a traditional Chinese medicine, which has been used as analgesic and sedative agents [2, 3]. Previous phytochemical studies led to the isolation of a series of aristolane, nardosinane, and guaiane sesquiterpenoids [3]. Aristolane is a rare class of natural sesquiterpenoid featuring a *gem*-dimethylcyclopropane moiety, and mainly isolated from the genus *Nardostachys* (terrestrial plants), *Laurencia* (marine organisms), *Axinyssa* (sponges), and *Russula* (fungi) [4].

**Fig. 1:**
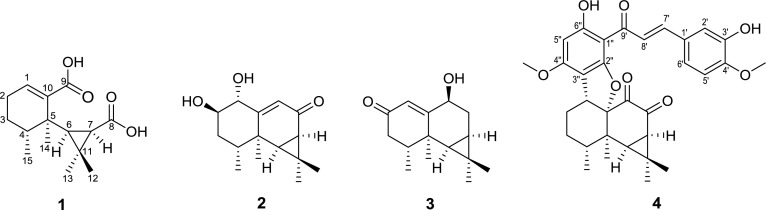
Structures of compounds **1**–**4**

As part of a BioBioPha [http://www.chemlib.cn] objective to assemble a large-scale natural product library valuable in the discovery of new drug leads from nature, the phytochemical investigation on the roots and rhizomes of *N. chinensis* resulted in the isolation of four new aristolane-type sesquiterpenoids, including one novel 8,9-secoaristolane, namely secoaristolenedioic acid (**1**), two aristolone derivatives, namely 1*α*,2*β*-dihydroxyaristolone (**2**), 9-epidebilon (**3**), and one rare aristolane-chalcone hybrid, namely 3′-hydroxynardoaristolone A (**4**) (Fig. 1), together with eight known aristolanes (aristolone [5], debilon [5], kanshone H [6], anthracophyllone [7], axinysone A [8], axinysone B [8], 1(10)-aristolen-2-one [9], and aristola-1(10),8-dien-2-one [10]), six know nardosinanes (nardosinone [11], nardosinonediol [11], desoxo-narchinol A [11], kanshone A [12], kanshone B [12], and narchinol B [13]), three known guaianes (isonardoperoxide [14], 10-epiteuclatriol [15], and aromadendrane-4*β*,10*α*-diol [16]), and three known eudesmanes (eudesma-3,11-dien-2-one [17], 7-epi-5-eudesmene-1*β*,11-diol [18], and nardoeudesmol A [6]). Herein, we describe the isolation and structure elucidation of these new compounds.

## Results and Discussion

**Fig. 2:**
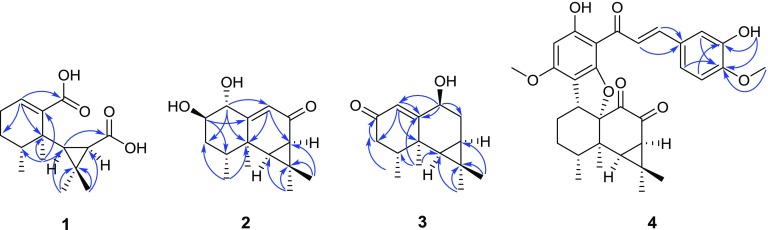
Key HMBC  correlations of **1**–**4**

**Fig. 3:**
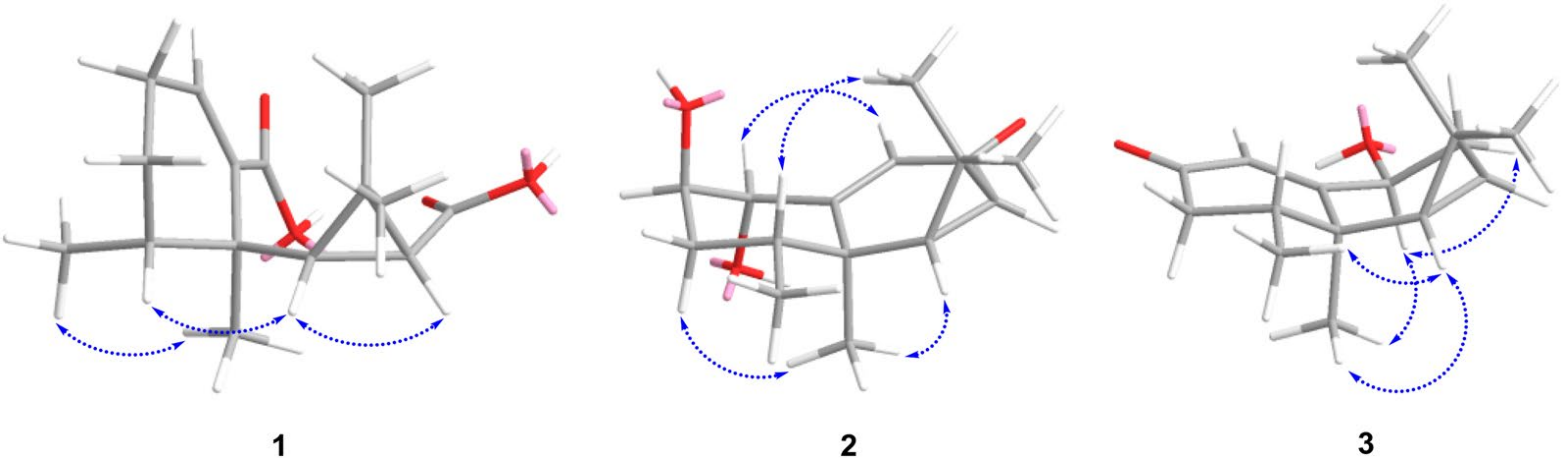
Key ROESY  correlations of **1**–**3**

Compound **1** was obtained as a white amorphous powder, and its molecular formula was determined to be C_15_H_22_O_4_ based on HRESIMS (pos.): *m/z* 289.1410 [M + Na]^+^ (calcd. for C_15_H_22_O_4_Na, 289.1410) with five degrees of unsaturation. The IR spectrum showed characteristic absorption bands for carbonyl functionalities (1687 cm^−1^). The ^1^H NMR spectrum (Table 1) in pyridine-*d*_5_ showed the signals for three tertiary methyls [*δ*_H_ 1.32, 1.81, and 1.91 (each 3H, s)], one secondary methyl [*δ*_H_ 0.95 (3H, d, *J* = 6.9 Hz)], two mutually coupled methine protons [*δ*_H_ 1.65 (1H, d, *J* = 9.8 Hz) and 1.92 (1H, d, *J* = 9.8 Hz)], and one olefinic proton [*δ*_H_ 6.96 (1H, t, *J* = 3.7 Hz)]. The ^13^C and DEPT spectra (Table 1) displayed a total of 15 carbon resonances, including four methyls, two methylenes, four methines (one olefinic at *δ*_C_ 135.1), and five quaternary carbons (two carbonyls at *δ*_C_ 170.8 and 175.3, one olefinic at *δ*_C_ 141.7). The cross-peaks of H-1/H-2, H-2/H-3, H-3/H-4, and H-6/H-7 in the ^1^H-^1^H COSY spectrum disclosed the following connectivities: C-1–C-2–C-3–C-4 and C-6–C-7. A *gem*-dimethylcyclopropane unit was deduced from the HMBC correlations (Fig. 2) from H_3_-12 (*δ*_H_ 1.91) to C-6 (*δ*_C_ 45.3), C-7 (*δ*_C_ 30.8), C-11 (*δ*_C_ 29.1), and C-13 (*δ*_C_ 32.4), from H_3_-13 (*δ*_H_ 1.32) to C-6, C-7, C-11, and C-12 (*δ*_C_ 16.9). Together with the HMBCs from H_3_-14 (*δ*_H_ 1.81) to C-4 (*δ*_C_ 37.0), C-5 (*δ*_C_ 40.8), and C-6, and from H_3_-15 (*δ*_H_ 0.95) to C-3 (*δ*_C_ 25.8), C-4, and C-5, an aristolane-type sesquiterpene skeleton was speculated for compound **1** [5]. A double bond located at C-1(10) was confirmed by the HMBCs from H_3_-14 (*δ*_H_ 1.81) to C-5 and C-10 (*δ*_C_ 141.7), and from H-1 (*δ*_H_ 6.96) to C-3 (*δ*_C_ 25.8) and C-5. Then, the presence of two newly formed carbonyl groups at C-8 and C-9 was determined by the HMBC correlations of H-1 (*δ*_H_ 6.96) to C-9 (*δ*_C_ 170.8) and H-6 (*δ*_H_ 1.65) to C-8 (*δ*_C_ 175.3), which indicated an oxidative cleavage had happened at C-8–C-9 in the ring B. The stereochemistry was defined by observation of the ROESY correlations between H-4/H-6 and H-6/H-7 (Fig. 3), consistent with that of the co-occurring aristolanes. Therefore, the structure of **1** was established as 8,9-seco-1(10)-aristolene-8,9-dioic acid and given the trivial name secoaristolenedioic acid. To our knowledge, this compound represents the first example of a secoaristolane.

**Table 1:** ^1^H and ^13^C NMR spectroscopic data of compounds **1**–**3**

	**1** ^a^		**1** ^b^		**2** ^b^		**3** ^c^	
No.	*δ*_H_	*δ*_C_	*δ*_H_	*δ*_C_	*δ*_H_	*δ*_C_	*δ*_H_	*δ*_C_
1	6.96 (t, 3.7)	135.1 (d)	6.65 (t, 3.9)	137.4 (d)	4.07 (dd, 3.0, 1.3)	77.3 (d)	6.12 (d, 1.8)	120.8 (d)
2α	2.09 (m)	23.2 (t)	2.09 (m)	24.1 (t)	3.91 (q-*like*, 2.9)	71.9 (d)		199.2 (s)
2β	2.09 (m)		2.14 (m)					
3α	1.90 (m)	25.8 (t)	1.83 (m)	26.5 (t)	1.99 (ddd, 14.4, 13.2, 2.3)	33.3 (t)	2.25 (m)	41.9 (t)
3β	1.41 (m)		1.44 (m)		1.53 (dddd, 14.4, 3.4, 3.4, 1.3)		2.23 (m)	
4	2.42 (m)	37.0 (d)	2.15 (m)	37.4 (d)	2.30 (m)	32.8 (d)	2.32 (m)	36.7 (d)
5		40.8 (s)		41.4 (s)		40.2 (s)		40.4 (s)
6	1.65 (d, 9.8)	45.3 (d)	1.36 (d, 9.8)	46.4 (d)	1.56 (d, 7.8)	42.2 (d)	0.67 (d, 9.1)	32.5 (d)
7	1.92 (d, 9.8)	30.8 (d)	1.41 (d, 9.8)	31.1 (d)	1.74 (dd, 7.8, 1.2)	37.6 (d)	0.93 (ddd, 9.6, 9.1, 4.0)	18.2 (d)
8α		175.3 (s)		176.1 (s)		199.7 (s)	2.49 (ddd, 13.7, 9.6, 7.6)	29.9 (t)
8β							1.45 (ddd, 13.7, 12.0, 4.0)	
9		170.8 (s)		171.8 (s)	5.87 (d, 1.2)	130.0 (d)	4.46 (ddd, 12.0, 7.6, 1.8)	67.2 (d)
10		141.7 (s)		141.3 (s)		168.3 (s)		174.7 (s)
11		29.1 (s)		30.3 (s)		27.2 (s)		19.2 (s)
12	1.91 (s)	16.9 (q)	1.48 (s)	16.6 (q)	1.25 (s)	16.3 (q)	0.95 (s)	17.3 (q)
13	1.32 (s)	32.4 (q)	1.17 (s)	32.6 (q)	1.23 (s)	30.2 (q)	1.02 (s)	29.1 (q)
14	1.81 (s)	19.8 (q)	1.32 (s)	20.3 (q)	1.35 (s)	25.0 (q)	1.25 (s)	22.6 (q)
15	0.95 (d, 6.9)	16.4 (q)	0.88 (d, 6.9)	16.6 (q)	1.08 (d, 7.0)	16.2 (q)	1.06 (d, 6.6)	15.3 (q)

^a,b,c^ Measured in pyridine-*d*_5_, CD_3_OD and CDCl_3_, respectively

**Fig. 4:**
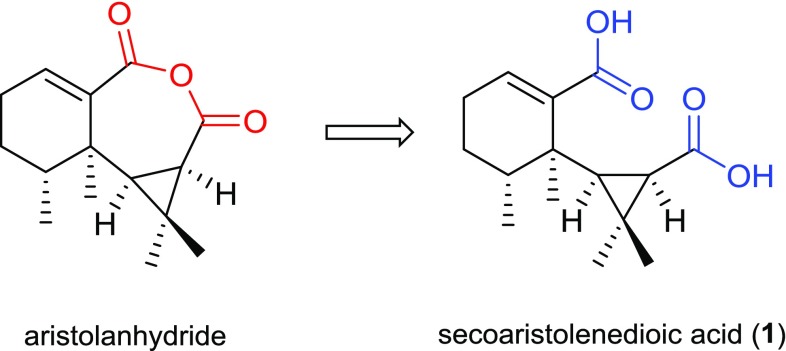
Structural revision of aristolanhydride

It was worth mentioning that the NMR data of compound **1** were almost identical to those of aristolanhydride (an unprecedented 8,9-cleavage anhydride derivative of aristolane-type sesquiterpene), recently isolated by Chen and co-workers from the same species [19]. Since the NMR spectra of aristolanhydride were initially measured in methanol-*d*_4_, we then re-tested the ^1^H and ^13^C-NMR of **1** using methanol-*d*_4_ and found that their NMR spectra were exactly the same. The structural difference between them was whether the dicarboxylic acid dehydrated to form a cyclic anhydride or not. Then we carefully examined the HRESIMS spectra of **1** and aristolanhydride. For compound **1**, a positive-ion HRESIMS exhibited an [M + Na]^+^ ion peak at *m/z* 289.1410, suggesting a molecular formula of C_15_H_22_O_4_ (calcd. for C_15_H_22_O_4_Na, 289.1410), while for aristolanhydride, a negative mode HRESIMS gave an [M − H]^−^ ion peak at *m/z* 265.1441, but Chen and co-workers mistakenly believed that it was an [M + OH]^−^ ion peak, and then led to a wrong dehydrated molecular formula C_15_H_20_O_3_. Furthermore, HPLC and TLC analysis were performed on an Agilent 1200 series HPLC system and silica gel F254 TLC plates, respectively, and a broad chromatogram peak and a long trailing spot (chloroform/methanol = 10/1) were observed, which also hinted the existence of carboxyl group not an anhydride. Above all, the structure of aristolanhydride should be revised as secoaristolenedioic acid (**1**) (Fig. 4).

Compound **2** was isolated as a white amorphous powder and possessed a molecular formula of C_15_H_22_O_3_ determined by positive HRESIMS: *m/z* 273.1462 [M + Na]^+^ (calcd. for C_15_H_22_O_3_Na, 273.1461) with five degrees of unsaturation. The IR spectrum showed characteristic absorption bands for hydroxy group (3445 cm^−1^) and conjugated carbonyl functionalities (1642 cm^−1^). The ^1^H NMR spectrum (Table 1) in methanol-*d*_4_ displayed a series of characteristic proton signals of an aristolane sesquiterpene: three tertiary methyls [*δ*_H_ 1.23, 1.25, and 1.35 (each 3H, s)], one secondary methyl [*δ*_H_ 1.08 (3H, d, *J* = 7.0 Hz)], two mutually coupled methine protons [*δ*_H_ 1.56 (1H, d, *J* = 7.8 Hz) and 1.74 (1H, dd, *J* = 7.8, 1.2 Hz)], and one olefinic protons [*δ*_H_ 5.87 (1H, d, *J* = 1.2 Hz)]. The ^13^C NMR and DEPT spectra (Table 1) showed a total of 15 carbon signals, characterized by four methyls, one methylene, six methines (including one olefinic at *δ*_C_ 130.0, two oxygenated at *δ*_C_ 71.9 and 77.3), and four quaternary carbons (including one keto carbonyl at *δ*_C_ 199.7 and one olefinic at *δ*_C_ 168.3). The above-mentioned NMR data revealed its structural features related to those of aristolone [5], with the major difference being the presence of two oxymethine groups at *δ*_H_ 3.91 (1H, q-*like*, *J* = 2.9 Hz)/*δ*_C_ 71.9 and *δ*_H_ 4.07 (1H, dd, *J* = 3.0, 1.3 Hz)/*δ*_C_ 77.3, indicative of the presence of two hydroxy groups. One of the hydroxy group located at C-1 was supported by the HMBC correlations (Fig. 2) from H-9 (*δ*_H_ 5.87) to C-1 (*δ*_C_ 77.3) and C-5 (*δ*_C_ 40.2), and from H-1 (*δ*_H_ 4.07) to C-5, C-9 (*δ*_C_ 130.0), and C-10 (*δ*_C_ 168.3), and the other was assigned to C-2 on the basis of the HMBCs from H-2 (*δ*_H_ 3.91) to C-4 (*δ*_C_ 32.8) and C-10. *α*-Axial orientation of the hydroxy at C-1 was deduced from the strong ROESY correlation of H-1/H-9 as well as the absence of correlation of H-1/H_3_-14 in the ROESY spectrum (Fig. 3). Moreover, the small *di*-equatorial couplings (*J*_*ee*_ = 3.0 Hz) of H_*eq*_-1/H_*eq*_-2, H_*eq*_-2/H_*eq*_-3 and the small axial-equatorial coupling (*J*_*ea*_ = 3.0 Hz) of H_*eq*_-2/H_*ax*_-3 revealed a *β*-axial orientation of the hydroxy at C-2. Hence, the structure of **2** was established as 1*α*,2*β*-dihydroxyaristolone.

Compound **3**, colorless oil, gave a molecular formula of C_15_H_22_O_2_ as determined by positive HRESIMS: *m/z* 257.1511 [M + Na]^+^ (calcd. for C_15_H_22_O_2_Na, 257.1512) with five degrees of unsaturation. The IR spectrum revealed the absorptions for hydroxy (3432 cm^−1^) and conjugated carbonyl groups (1646 cm^−1^). The NMR spectra (Table 1) in chloroform-*d* also exhibited a series of characteristic signals due to an aristolane sesquiterpene: three tertiary methyls [*δ*_H_ 0.95, 1.02, and 1.25 (each 3H, s)], one secondary methyl [*δ*_H_ 1.06 (3H, d, *J* = 6.6 Hz)], two mutually coupled up-field methine protons [*δ*_H_ 0.67 (1H, d, *J* = 9.1 Hz) and 0.93 (1H, ddd, *J* = 9.6, 9.1, 4.0 Hz)], and one olefinic proton [*δ*_H_ 6.12 (1H, d, *J* = 1.8 Hz)] in the ^1^H NMR spectrum; a total of 15 carbon resonances classified as four methyls, two methylenes, five methines (including one olefinic at *δ*_C_ 120.8 and one oxygenated at *δ*_C_ 67.2), and four quaternary carbons (including one keto carbonyl at *δ*_C_ 199.2 and one olefinic at *δ*_C_ 174.7) in the ^13^C and DEPT spectra. The above NMR spectroscopic features were highly similar to those of debilon [9*α*-hydroxy-1(10)-aristolen-2-one], with the obvious difference being the chemical shift of the oxymethine [5]. The hydroxy was assigned to C-9 according to the HMBC correlations (Fig. 2) from H-1 (*δ*_H_ 6.12) to C-9 (*δ*_C_ 67.2), from H-9 (*δ*_H_ 4.46) to C-1 (*δ*_C_ 120.8), C-8 (*δ*_C_ 29.9), and C-10 (*δ*_C_ 174.7), which suggested that compound **3** shared the same planar structure with debilon. The strong ROESY correlation of H-9/H_3_-14 (Fig. 3), together with the *di*-axial coupling (*J* = 12.0 Hz) of H_*ax*_-9/H_*ax*_-8 and axial-equatorial coupling (*J* = 7.6 Hz) of H_*ax*_-9/H_*eq*_-8, suggested a *β*-equatorial orientation of the hydroxy group at C-9. Therefore, compound **3** was established as 9*β*-hydroxy-1(10)-aristolen-2-one and named as 9-epidebilon.

Compound **4** was isolated as an orange amorphous powder, with a molecular formula of C_32_H_34_O_8_ according to the positive HRESIMS: *m/z* 569.2147 [M + Na]^+^ (calcd. for C_32_H_34_O_8_Na, 569.2146). In the NMR spectra (Table 2), three tertiary methyls [*δ*_H_ 1.30, 1.31, and 1.37 (each 3H, s)], one secondary methyl [*δ*_H_ 1.08 (3H, d, *J* = 6.5 Hz)], two mutually coupled methine doublets [*δ*_H_ 1.49 (1H, d, *J* = 7.8 Hz) and 2.51 (1H, d, *J* = 7.8 Hz)], together with 15 carbon resonances (four methyls, two methylenes, four methines, and five quaternary carbons) were recognized as an aristolane structural unit. Moreover, a *tran*s-*α*,*β*-unsaturated ketone group [*δ*_H_ 7.78 (1H, d, *J* = 15.4 Hz)/*δ*_C_ 123.9; *δ*_H_ 7.73 (1H, d, *J* = 15.4 Hz)/*δ*_C_ 143.4; *δ*_C_ 190.8], a chelated hydroxy [*δ*_H_ 13.99 (1H, s)] and one aromatic proton [*δ*_H_ 6.05 (1H, s)] due to a pentasubstituted phenyl ring, as well as one typical ABX system [*δ*_H_ 7.37 (1H, d, *J* = 2.0 Hz), 6.88 (1H, d, *J* = 8.3 Hz), and 7.09 (1H, dd, *J* = 8.3, 2.0 Hz)] were observed, which could be recognized as a chalcone structural unit. The above-mentioned NMR data were very close to those of nardoaristolone A, an unprecedented aristolane-chalcone hybrid recently isolated from the same species [20], and were almost identical in the aristolane part. The prominent difference between the two compounds came from the substituted pattern of ring B in chalcone part. The presence of a 1,2,4-trisubtituted ring B in **4**, instead of *p*-substituted in nardoaristolone A, was confirmed from the HMBC correlations (Fig. 2) from H-2′ (*δ*_H_ 7.37) to C-4′ (*δ*_C_ 149.0) and C-6′ (*δ*_C_ 123.7), from H-5′ (*δ*_H_ 6.88) to C-1′ (*δ*_C_ 129.1) and C-3′ (*δ*_C_ 146.0), and from H-6′ (*δ*_H_ 7.09) to C-2′ (*δ*_C_ 112.1) and C-4′. Also a newly emerged hydroxy [*δ*_H_ 5.85 (1H, s)] and a methoxy group [*δ*_H_ 3.94 (3H, s)] located at C-3′ and C-4′, respectively, were deduced from the HMBC correlations of the hydroxy signal (*δ*_H_ 5.85) to C-3′ and the methoxy signal (*δ*_H_ 3.94) to C-4′, together with the ROESY correlations of H-2′↔OH-3′ and H-5′↔OCH_3_-4′. Hence, the structure of **4** was established and named as 3′-hydroxynardoaristolone A. To our knowledge, only the two aristolane-chalcone hybrids (nardoaristolone A and 3′-hydroxynardoaristolone A) were reported until now.

**Table 2:** ^1^H and ^13^C NMR spectroscopic data of compound **4** in CDCl_3_

No.	*δ*_H_	*δ*_C_	No.	*δ*_H_	*δ*_C_
1	4.09 (dd, 10.2, 7.3)	35.5 (d)	1′		129.1 (s)
2	1.87 (m)	22.8 (t)	2′	7.37 (d, 2.0)	112.1 (d)
	2.21 (m)		3′		146.0 (s)
3	1.38 (m)	25.6 (t)	4′		149.0 (s)
	1.76 (m)		5′	6.88 (d, 8.3)	110.6 (d)
4	1.80 (m)	31.6 (d)	6′	7.09 (dd, 8.3, 2.0)	123.7 (d)
5		44.4 (s)	7′	7.73 (d, 15.4)	143.4 (d)
6	1.49 (d, 7.8)	42.2 (d)	8′	7.78 (d, 15.4)	123.9 (d)
7	2.51 (d, 7.8)	39.3 (d)	9′		190.8 (s)
8		197.3 (s)	1′′		102.7 (s)
9		195.7 (s)	2′′		157.6 (s)
10		99.1 (s)	3′′		109.4 (s)
11		31.5 (s)	4′′		162.0 (s)
12	1.31 (s)	17.8 (q)	5′′	6.05 (s)	94.0 (d)
13	1.30 (s)	31.1 (q)	6′′		167.3 (s)
14	1.37 (s)	19.8 (q)	3′-OH	5.85 (s)	
15	1.08 (d, 6.5)	16.4 (q)	6′′-OH	13.99 (s)	
			4′-OCH_3_	3.94 (s)	56.0 (q)
			4′′-OCH_3_	3.82 (s)	55.7 (q)

## Experimental Section

### General Experimental Procedures

Optical rotations were measured on a Jasco P-1020 automatic digital polarimeter. UV data were obtained from HPLC online analysis. IR spectra (KBr) were obtained on a Bruker Tensor-27 infrared spectrophotometer. NMR spectra were carried out on a Bruker Avance III 600 or Bruker DRX-500 spectrometer with deuterated solvent signals used as internal standards. ESIMS and HRESIMS were measured using an Agilent G6230 time-of-flight mass spectrometer. MPLC was performed on a Büchi apparatus equipped with Büchi fraction collector C-660, Büchi pump module C-605 and manager C-615. Preparative HPLC separation was performed using an Agilent 1260 series HPLC system equipped with a Zorbax SB-C_18_ column (5 *μ*m, 21.2 × 150 mm). Silica gel (200–300 mesh, Qingdao Marine Chemical Inc., China), MCI gel CHP-20P (75–150 *μ*m, Mitsubishi Chemical Corporation, Japan), Chromatorex C_18_ (40–75 *μ*m, Fuji Silysia Chemical Ltd., Japan) and Sephadex LH-20 (GE Healthcare Bio-Sciences AB, Uppsala, Sweden) were used for column chromatography. Fractions were monitored and analyzed using TLC, in combination with Agilent 1200 series HPLC system equipped by an Extend-C_18_ column (5 *μ*m, 4.6 × 150 mm).

### Plant Material

The roots and rhizomes of *Nardostachys chinensis* were purchased from Luosiwan Chinese herbal medicine market in Kunming, People’s Republic of China, in August 2015 and identified by Mr. Yu Chen of Kunming Institute of Botany, Chinese Academy of Sciences. A voucher specimen (No. BBP0656026NC) was deposited at BioBioPha Co., Ltd.

### Extraction and Isolation

The air-dried and powdered roots and rhizomes of *N. chinensis* (12.0 kg) were extracted with 95% aqueous EtOH (3 × 18 L) at room temperature to obtain a crude extract (900 g) after evaporation of the solvent. The crude extract was fractionated by silica gel column chromatography successively eluted with a gradient of increasing acetone in petroleum ether (PE) (10:1, 8:1, 6:1, 5:1, 2:1, 1:1, 0:1; v/v) and then methanol to afford fractions A–H, respectively. Fraction E was separated by Sephadex LH-20 (CHCl_3_/MeOH, 1:1; *v*/*v*), MPLC (MeOH/H_2_O; 20% → 30%), silica gel (CHCl_3_/acetone, 10:1; *v*/*v*), and prep-HPLC (MeOH/H_2_O; 51%) to yield compound **3** (70 mg). Fraction F was separated by silica gel (CHCl_3_/acetone; 100:1 → 50:1), Sephadex LH-20 (MeOH), and prep-HPLC (MeOH/H_2_O; 48%) to yield compounds **1** (47 mg) and **4** (31 mg). Fraction G was further isolated sequentially using Sephadex LH-20 (MeOH), prep-MPLC (MeOH/H_2_O; 0 → 100%), and prep-HPLC (MeOH/H_2_O; 55%) to afford compound **2** (14 mg). The retention times (*t*_R_) of **1**–**4** on an analytical HPLC Thermo Hypersil BDS C_18_ column (20% → 100% MeOH in H_2_O over 8.0 min followed by 100% MeOH to 13.0 min, 1.0 ml/min, 22 °C) were 8.51, 7.25, 8.68, and 10.37 min, respectively.

### Secoaristolenedioic Acid (**1**)

White amorphous powder; [*α*]_D_^25^ −104.0 (*c* 0.20, MeOH); UV (MeOH) *λ*_max_: 215 nm; IR (KBr) *ν*_max_: 3443, 3428, 2921, 1687, 1676, 1638, 1446, 1417, 1384, 1294 cm^−1^; ^1^H and ^13^C NMR data: see Table 1; ESIMS (pos.): *m/z* 289 [M + Na]^+^; HRESIMS (pos.): *m/z* 289.1410 [M + Na]^+^ (calcd. for C_15_H_22_O_4_Na, 289.1410).

### 1*α*,2*β*-Dihydroxyaristolone (**2**)

White amorphous powder; [*α*]_D_^25^ −245.3 (*c* 0.20, MeOH); UV (MeOH) *λ*_max_: 235 nm; IR (KBr) *ν*_max_: 3445, 2966, 2922, 1642, 1449, 1383, 1054 cm^−1^; ^1^H and ^13^C NMR data: see Table 1; ESIMS (pos.): *m/z* 273 [M + Na]^+^; HRESIMS (pos.): *m/z* 273.1462 [M + Na]^+^ (calcd. for C_15_H_22_O_3_Na, 273.1461).

### 9-Epidebilon (**3**)

Colorless oil; [*α*]25 D +14.0 (*c* 0.20, MeOH); UV (MeOH) *λ*_max_: 242 nm; IR (KBr) *ν*_max_: 3432, 2967, 2942, 2882, 1710, 1646, 1455, 1383, 1193, 1053 cm^−1^; ^1^H and ^13^C NMR data: see Table 1; ESIMS (pos.): *m/z* 257 [M + Na]^+^; HRESIMS (pos.): *m/z* 257.1511 [M + Na]^+^ (calcd. for C_15_H_22_O_2_Na, 257.1512).

### 3′-Hydroxynardoaristolone A (**4**)

Orange amorphous powder; [*α*]_D_^25^ +223.7 (*c* 0.20, MeOH); UV (MeOH) *λ*_max_: 262, 315 (sh), 379 nm; IR (KBr) *ν*_max_: 3443, 2960, 2930, 1632, 1589, 1560, 1509, 1441, 1270, 1223, 1030 cm^−1^; ^1^H and ^13^C NMR data: see Table 2; ESIMS (pos.): *m/z* 569 [M + Na]^+^; HRESIMS (pos.): *m/z* 569.2147 [M + Na]^+^ (calcd. for C_32_H_34_O_8_Na, 569.2146).

## Electronic supplementary material

Below is the link to the electronic supplementary material.

Supplementary material 1 (DOC 1350 kb)
