# Supplementary material for: Aristolane-type Sesquiterpenoids from Nardostachys chinensis and Revised Structure of Aristolanhydride
Source: Nat Prod Bioprospect. 2019 Mar 8;9(2):149–55. doi: 10.1007/s13659-019-0200-7 (PMC6426942; doi:10.1007/s13659-019-0200-7)

**Electronic Supplementary Material**

**Aristolane-type Sesquiterpenoids from *Nardostachys chinensis* and Revised Structure of Aristolanhydride**

**Li-Xia Wang ^1,2^** **• Xian-Jun Jiang ^1,2^ • Xiang-Mei Li ^1,2^ • Mei-Fen Mao ^1,2^ • Guo-Zhu Wei ^1,2^ • Fei Wang ^1,2^**

Structures of compounds **1**–**4**

*To whom correspondence should be addressed.

E-mail: [f.wang@mail.biobiopha.com](mailto:f.wang@mail.biobiopha.com)

**Content list**

**S1.** ^1^H NMR spectrum (500 MHz, pyridine-*d*_5_) of secoaristolenedioic acid (**1**).

**S2.** ^13^C NMR spectrum (125 MHz, pyridine-*d*_5_) of secoaristolenedioic acid (**1**).

**S3.** HMBC spectrum (500 MHz, pyridine-*d*_5_) of secoaristolenedioic acid (**1**).

**S4.** HSQC spectrum (500 MHz, pyridine-*d*_5_) of secoaristolenedioic acid (**1**).

**S5.** ROESY spectrum (500 MHz, pyridine-*d*_5_) of secoaristolenedioic acid (**1**).

**S6.** ^1^H NMR spectrum (500 MHz, CD_3_OD) of secoaristolenedioic acid (**1**).

**S7.** ^13^C NMR spectrum (125 MHz, CD_3_OD) of secoaristolenedioic acid (**1**).

**S8.** IR spectrum of secoaristolenedioic acid (**1**).

**S9.** HR-ESI-MS spectrum of secoaristolenedioic acid (**1**).

**S10.** HR-ESI-MS spectrum of aristolanhydride [19]

**S11.** ^1^H NMR spectrum (500 MHz, CD_3_OD) of 1*α*,2*β*-dihydroxyaristolone (**2**).

**S12.** ^13^C NMR spectrum (125 MHz, CD_3_OD) of 1*α*,2*β*-dihydroxyaristolone (**2**).

**S13.** HMBC spectrum (500 MHz, CD_3_OD) of 1*α*,2*β*-dihydroxyaristolone (**2**).

**S14.** HSQC spectrum (500 MHz, CD_3_OD) of 1*α*,2*β*-dihydroxyaristolone (**2**).

**S15.** ROESY spectrum (500 MHz, CD_3_OD) of 1*α*,2*β*-dihydroxyaristolone (**2**).

**S16.** ^1^H NMR spectrum (500 MHz, CDCl_3_) of 9-epidebilon (**3**).

**S17.** ^13^C NMR spectrum (125 MHz, CDCl_3_) of 9-epidebilon (**3**).

**S18.** HMBC spectrum (500 MHz, CDCl_3_) of 9-epidebilon (**3**).

**S19.** HSQC spectrum (500 MHz, CDCl_3_) of 9-epidebilon (**3**).

**S20.** ROESY spectrum (500 MHz, CDCl_3_) of 9-epidebilon (**3**).

**S21.** ^1^H NMR spectrum (500 MHz, CDCl_3_) of 3′-hydroxynardoaristolone A **(4)**.

**S22.** ^13^C NMR spectrum (125 MHz, CDCl_3_) of 3′-hydroxynardoaristolone A **(4)**.

**S23.** HMBC spectrum (500 MHz, CDCl_3_) of 3′-hydroxynardoaristolone A **(4)**.

**S24.** HSQC spectrum (500 MHz, CDCl_3_) of 3′-hydroxynardoaristolone A **(4)**.

**S25.** ROESY spectrum (500 MHz, CDCl_3_) of 3′-hydroxynardoaristolone A **(4)**.

**S1.** ^1^H NMR spectrum (500 MHz, pyridine-*d*_5_) of secoaristolenedioic acid (**1**).


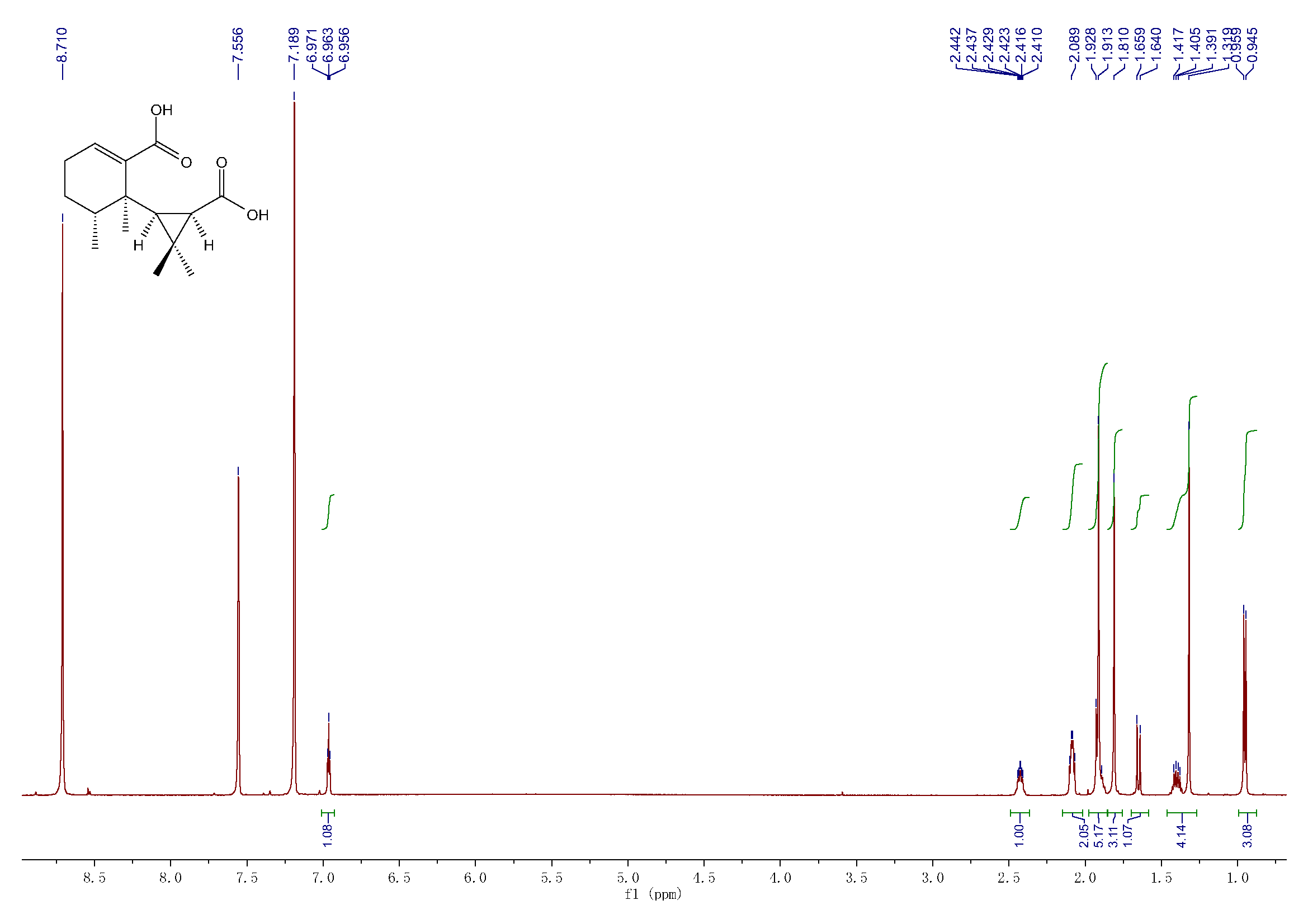


**S2.** ^13^C NMR spectrum (125 MHz, pyridine-*d*_5_) of secoaristolenedioic acid (**1**).


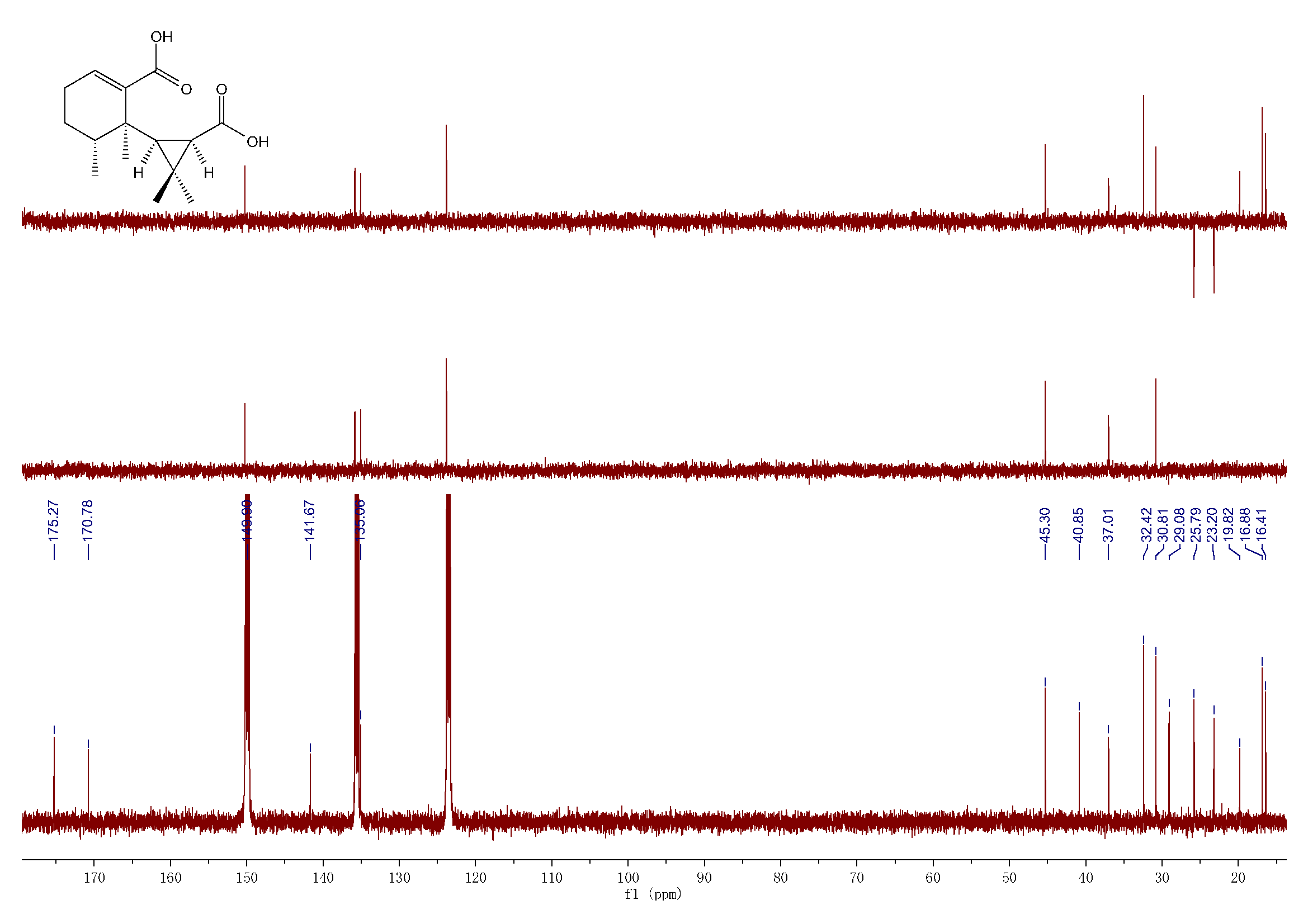


**S3.** HMBC spectrum (500 MHz, pyridine-*d*_5_) of secoaristolenedioic acid (**1**).


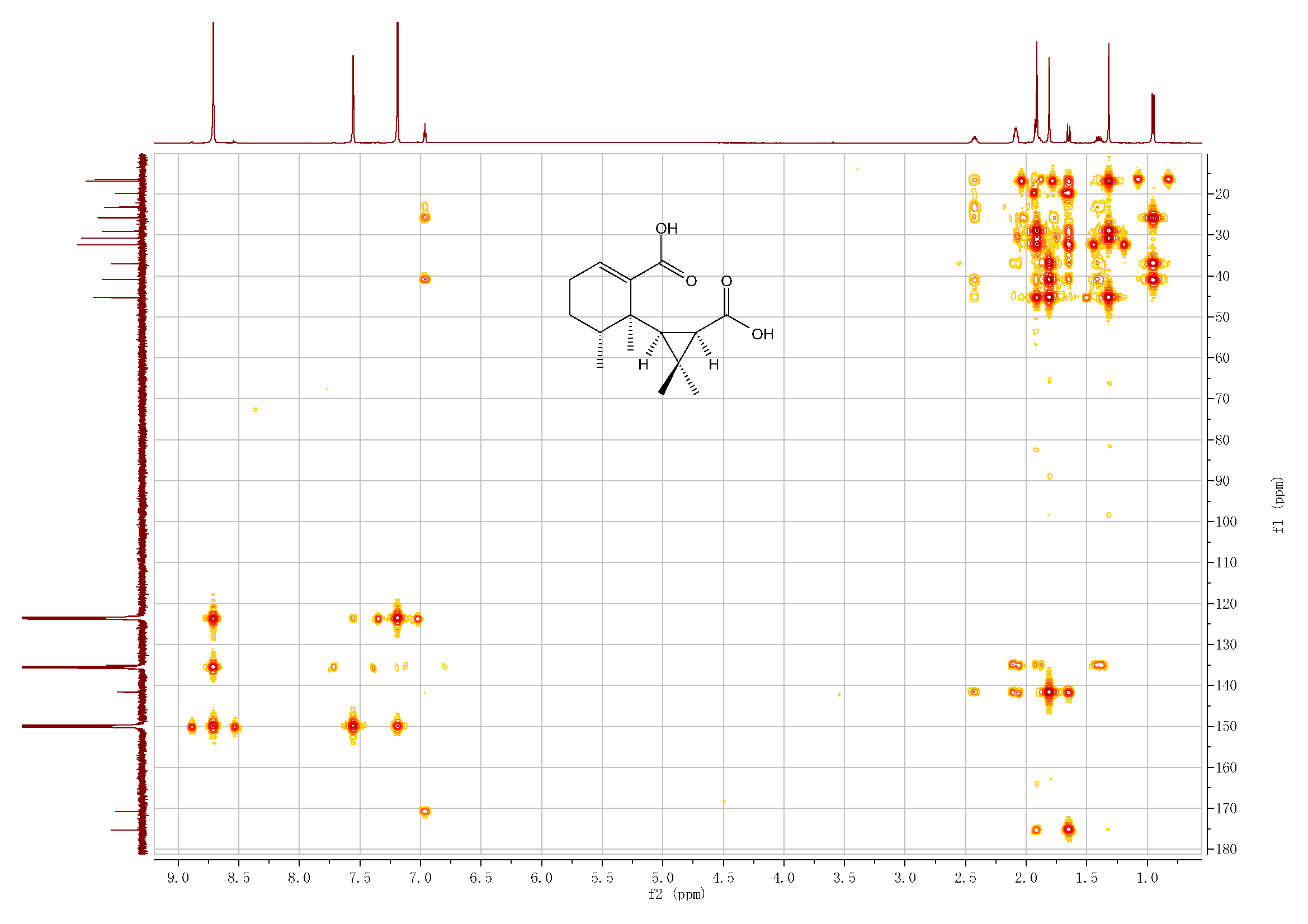


**S4.** HSQC spectrum (500 MHz, pyridine-*d*_5_) of secoaristolenedioic acid (**1**).


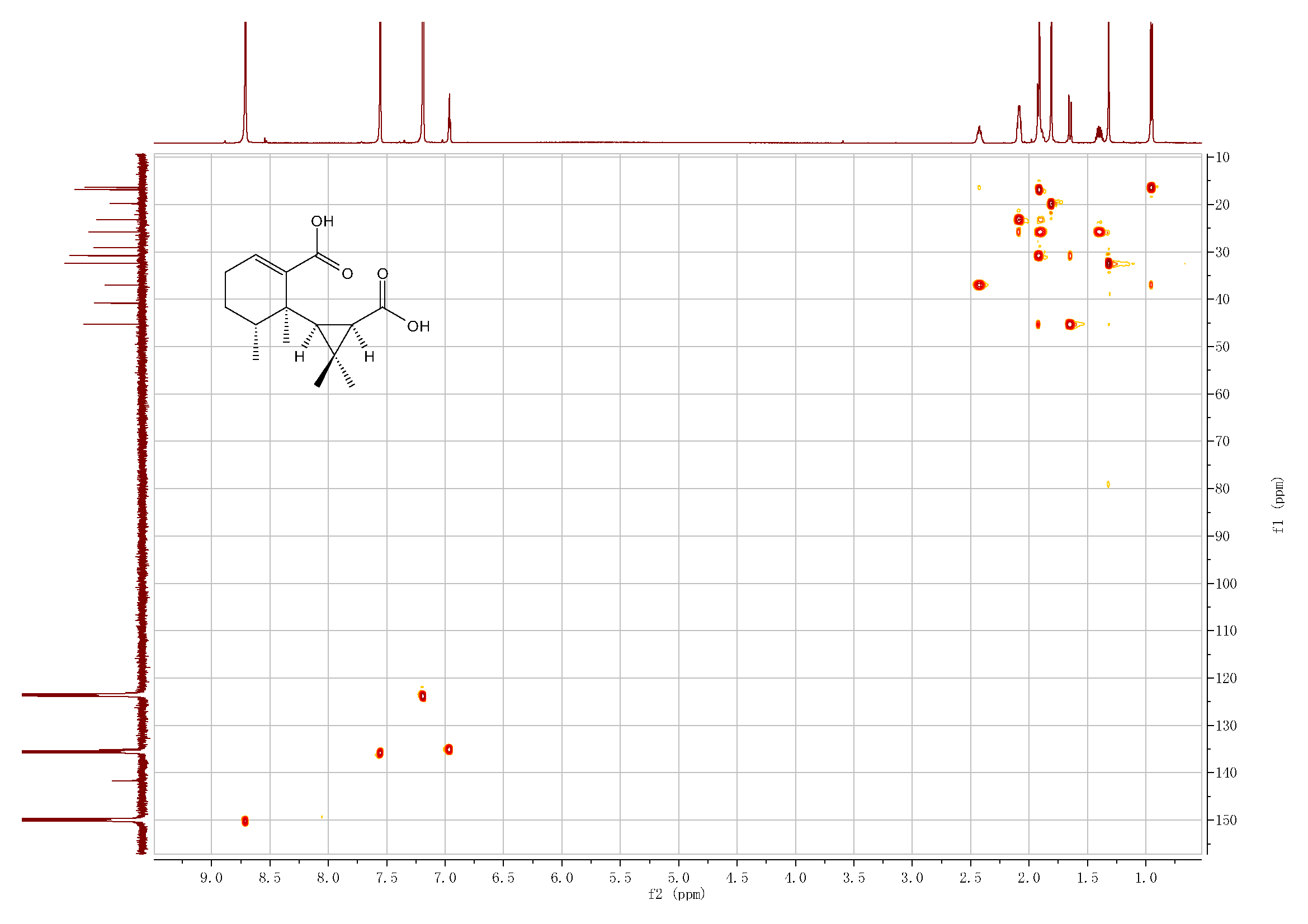


**S5.** ROESY spectrum (500 MHz, pyridine-*d*_5_) of secoaristolenedioic acid (**1**).


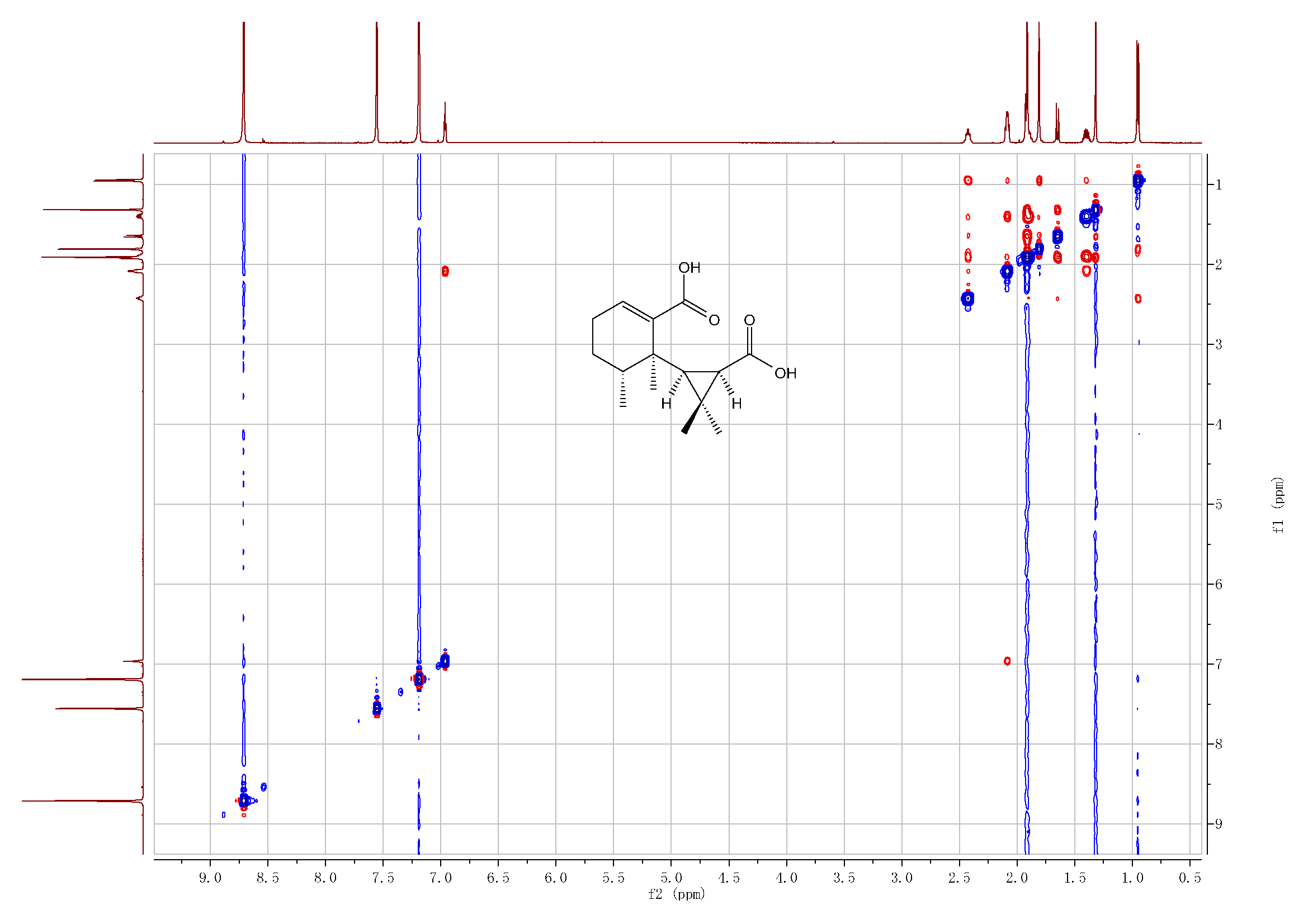


**S6.** ^1^H NMR spectrum (500 MHz, CD_3_OD) of secoaristolenedioic acid (**1**).


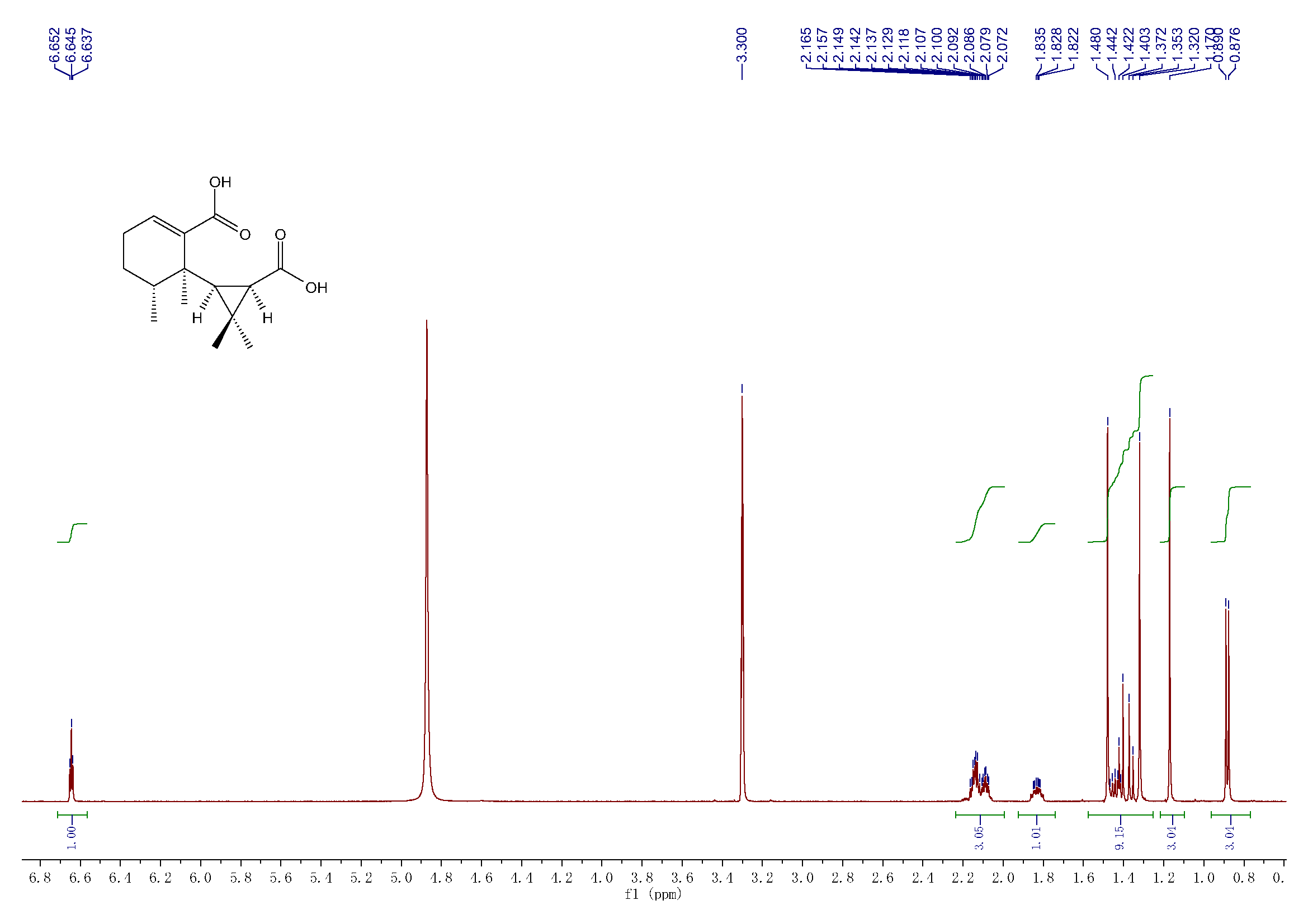


**S7.** ^13^C NMR spectrum (125 MHz, CD_3_OD) of secoaristolenedioic acid (**1**).


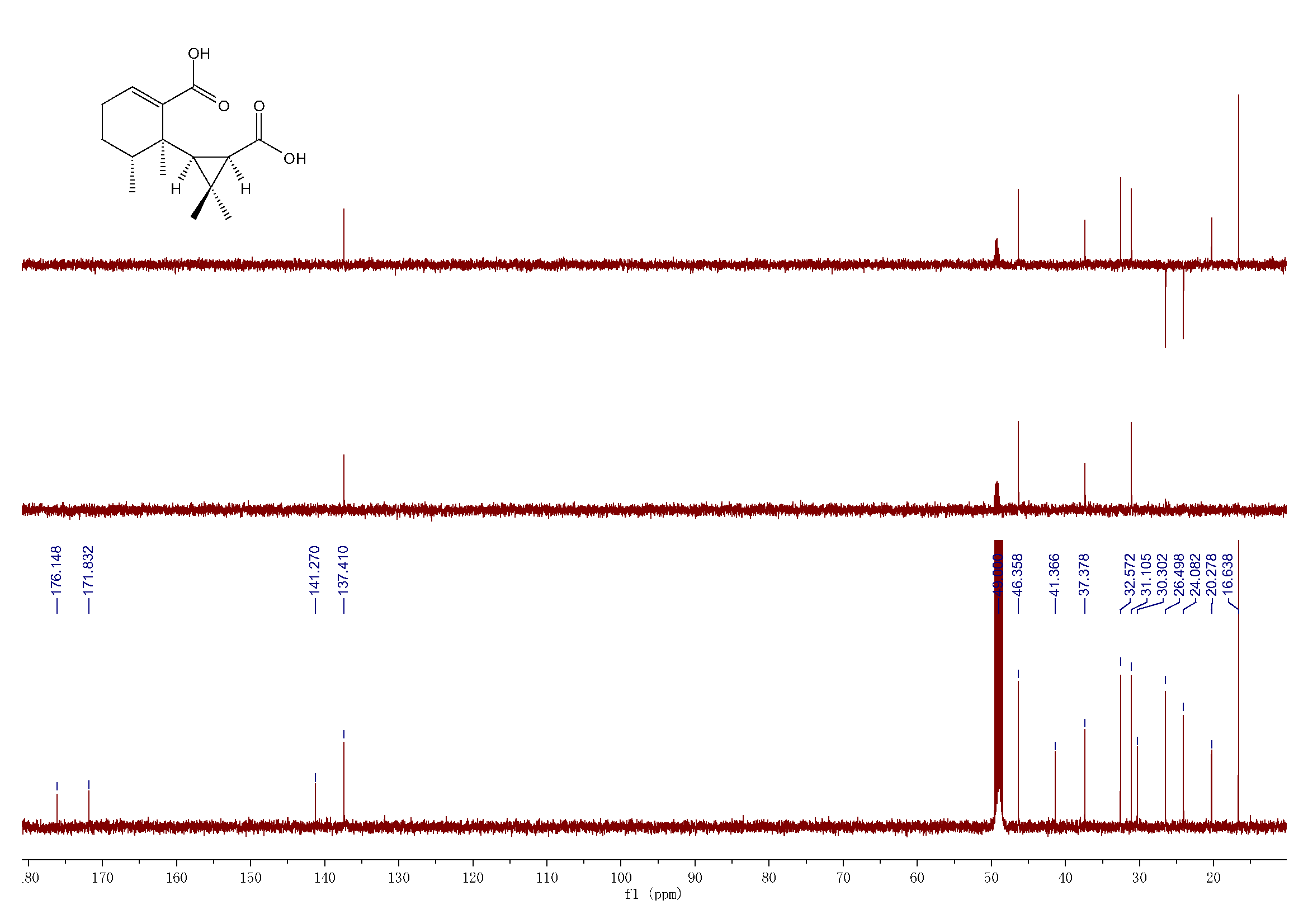


**S8.** IR spectrum of secoaristolenedioic acid (**1**).

**
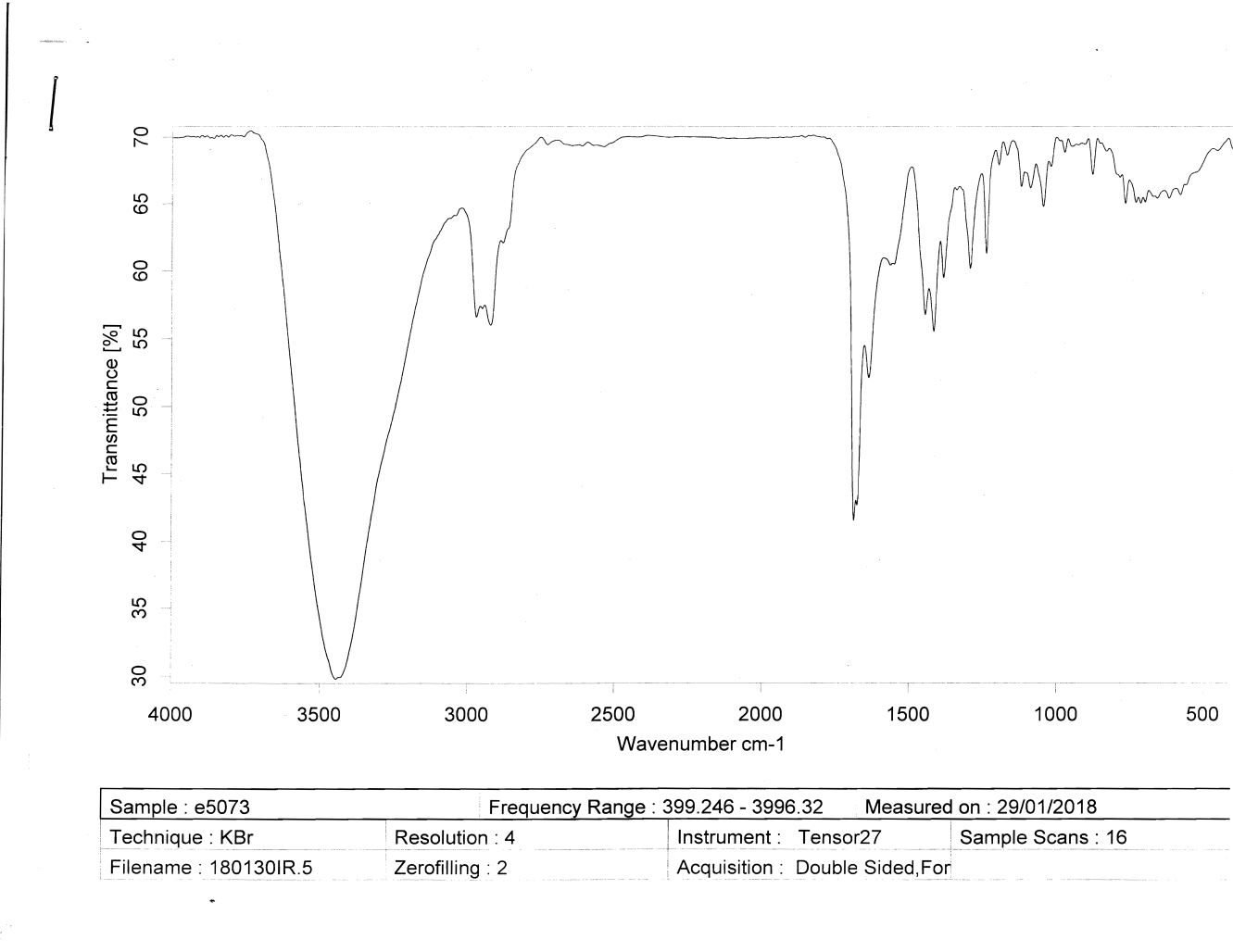
**

**S9.** HR-ESI-MS spectrum of secoaristolenedioic acid (**1**).

**
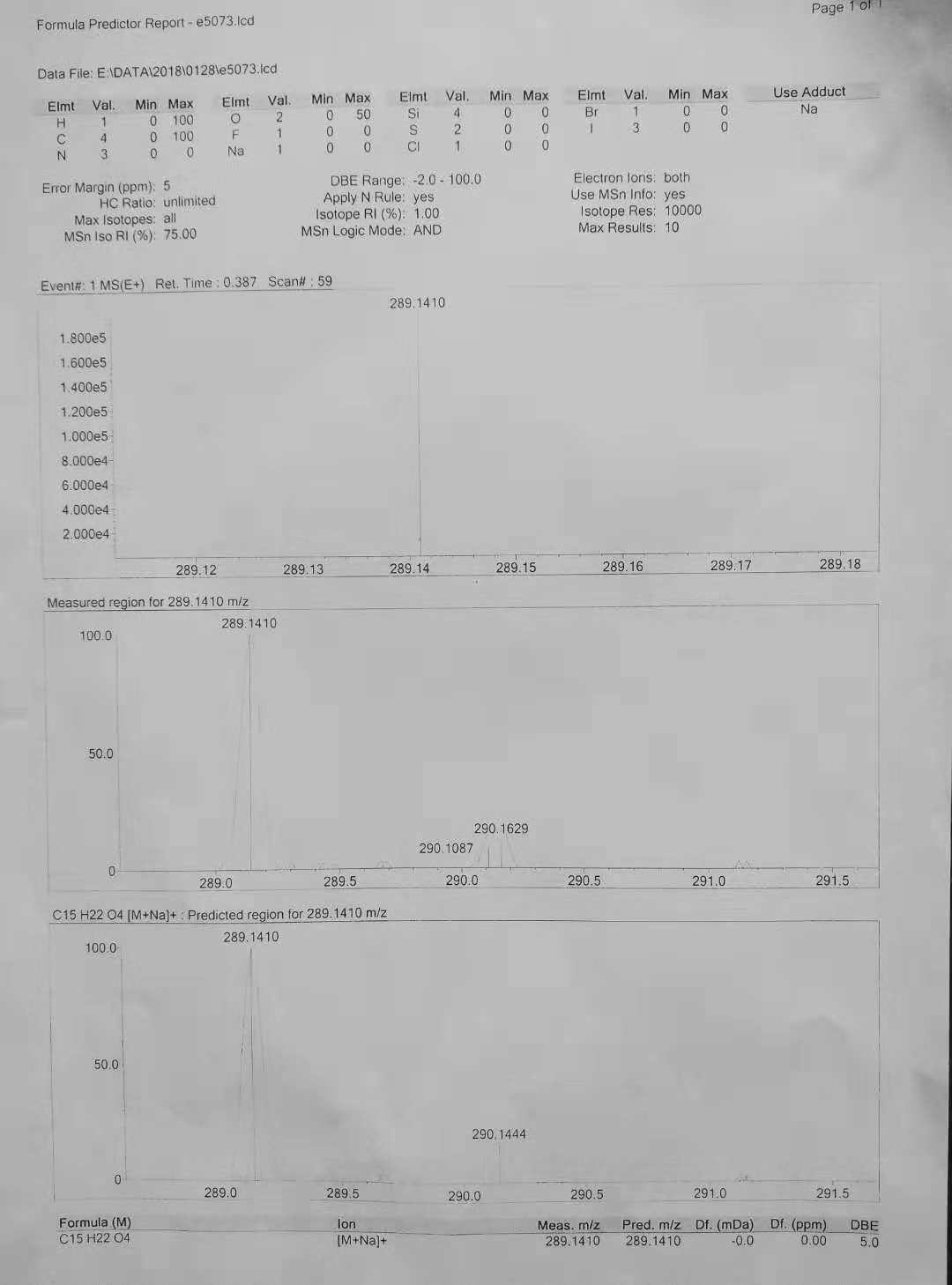
**

**S10.** HR-ESI-MS spectrum of aristolanhydride [19]


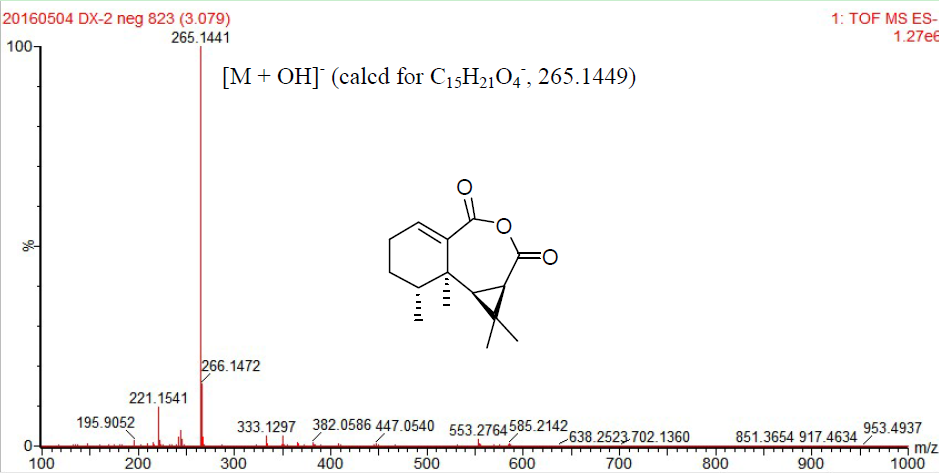


**S11.** ^1^H NMR spectrum (500 MHz, CD_3_OD) of 1*α*,2*β*-dihydroxyaristolone (**2**).


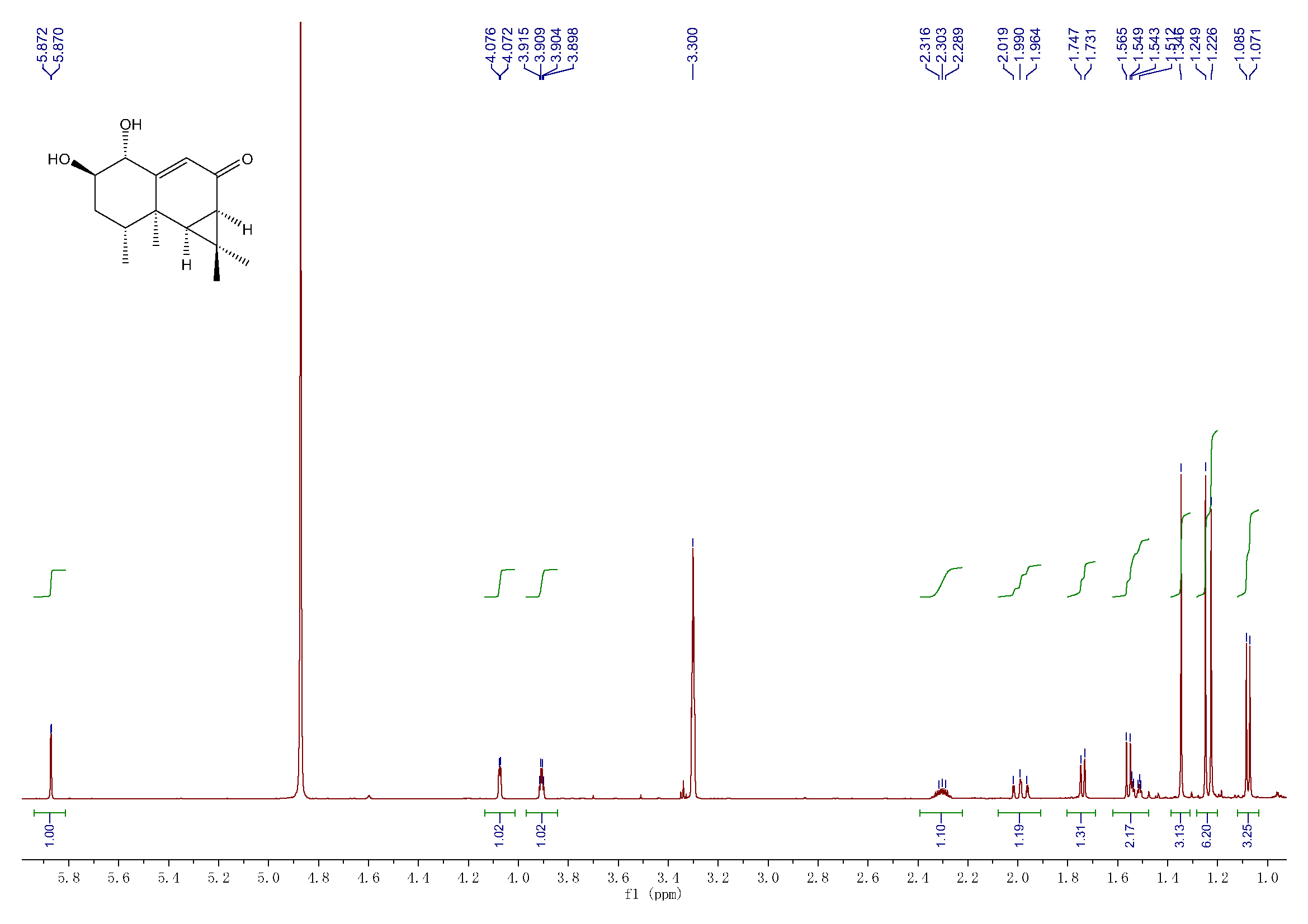


**S12.** ^13^C NMR spectrum (125 MHz, CD_3_OD) of 1*α*,2*β*-dihydroxyaristolone (**2**).


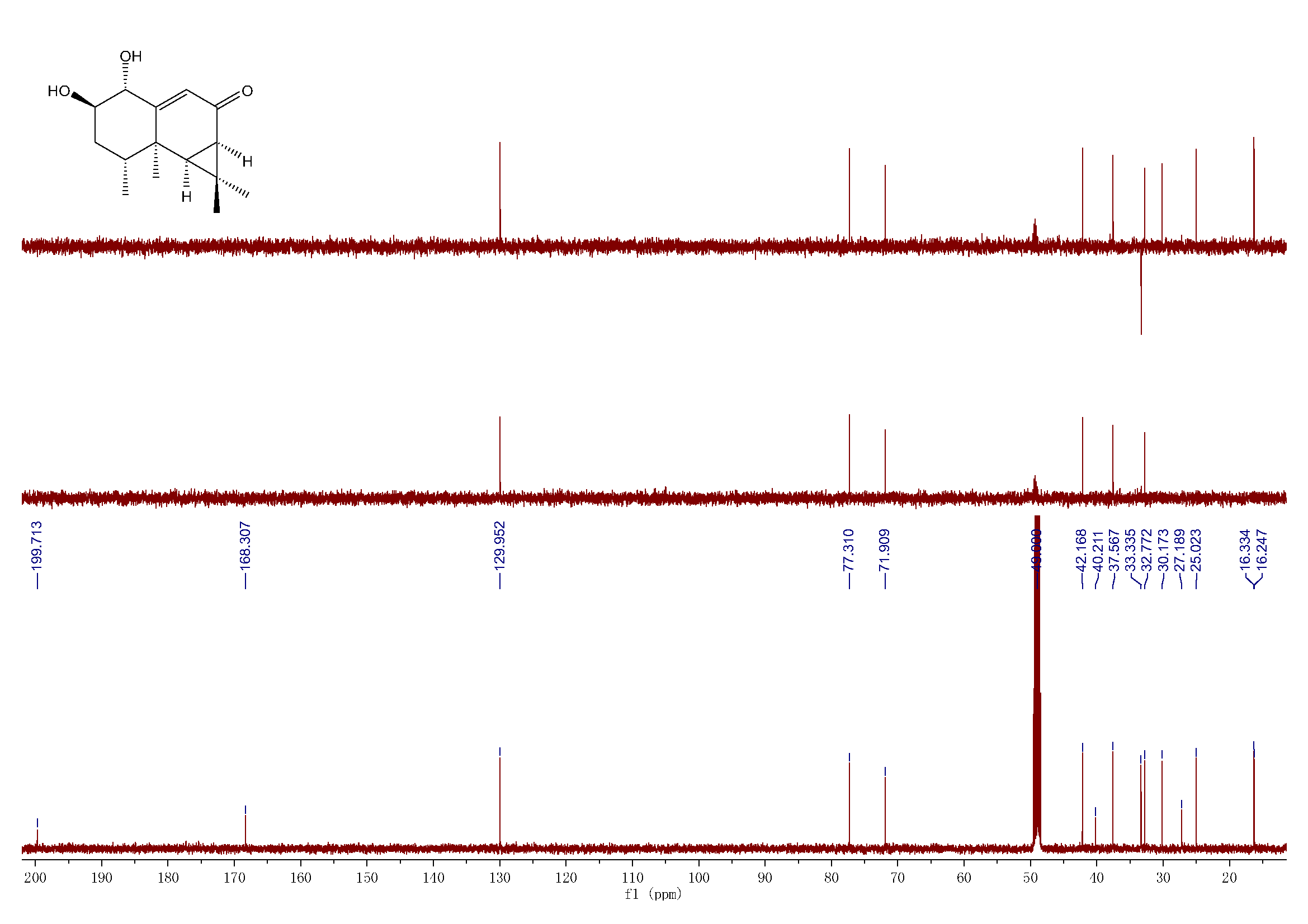


**S13.** HMBC spectrum (500 MHz, CD_3_OD) of 1*α*,2*β*-dihydroxyaristolone (**2**).


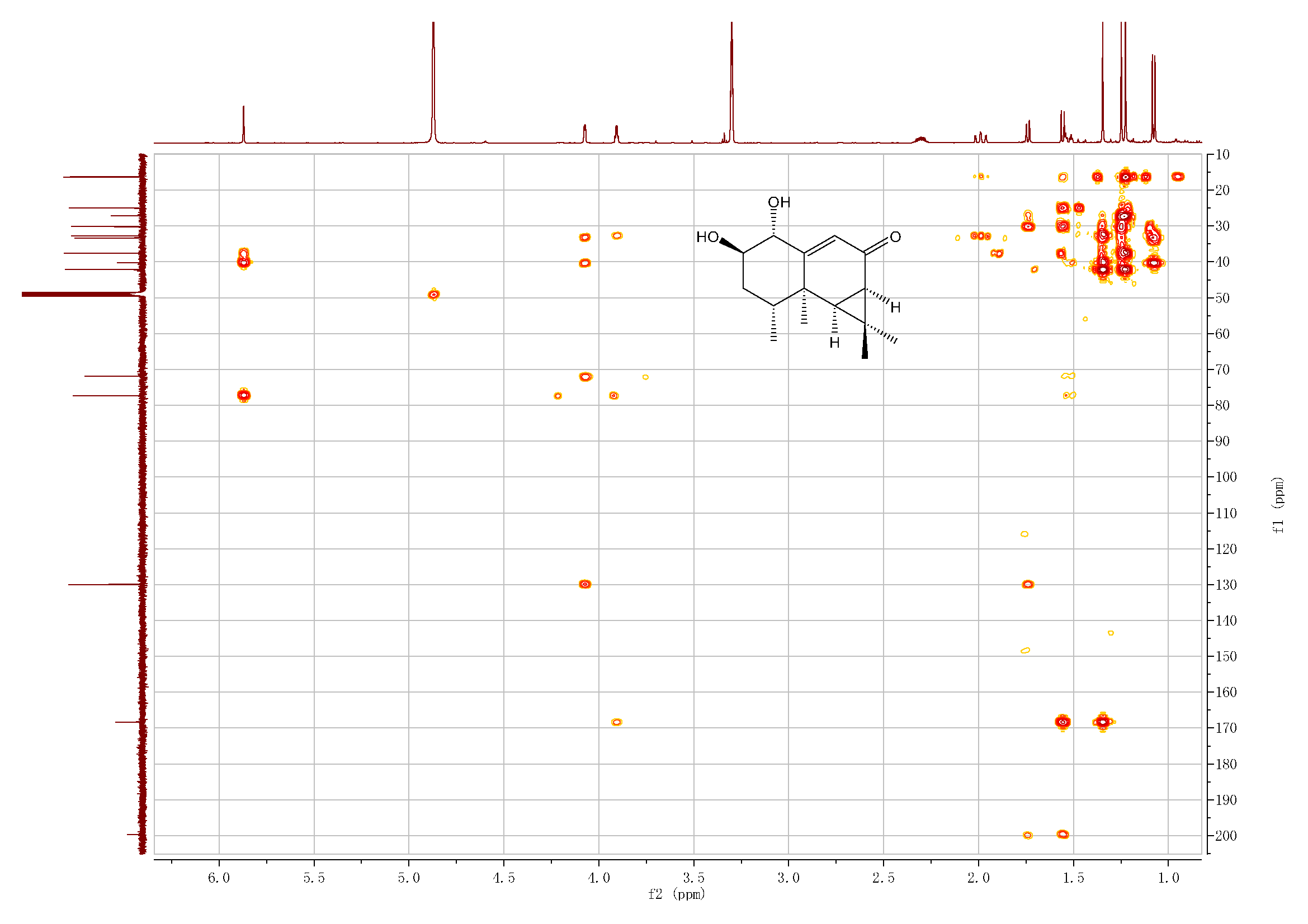


**S14.** HSQC spectrum (500 MHz, CD_3_OD) of 1*α*,2*β*-dihydroxyaristolone (**2**).


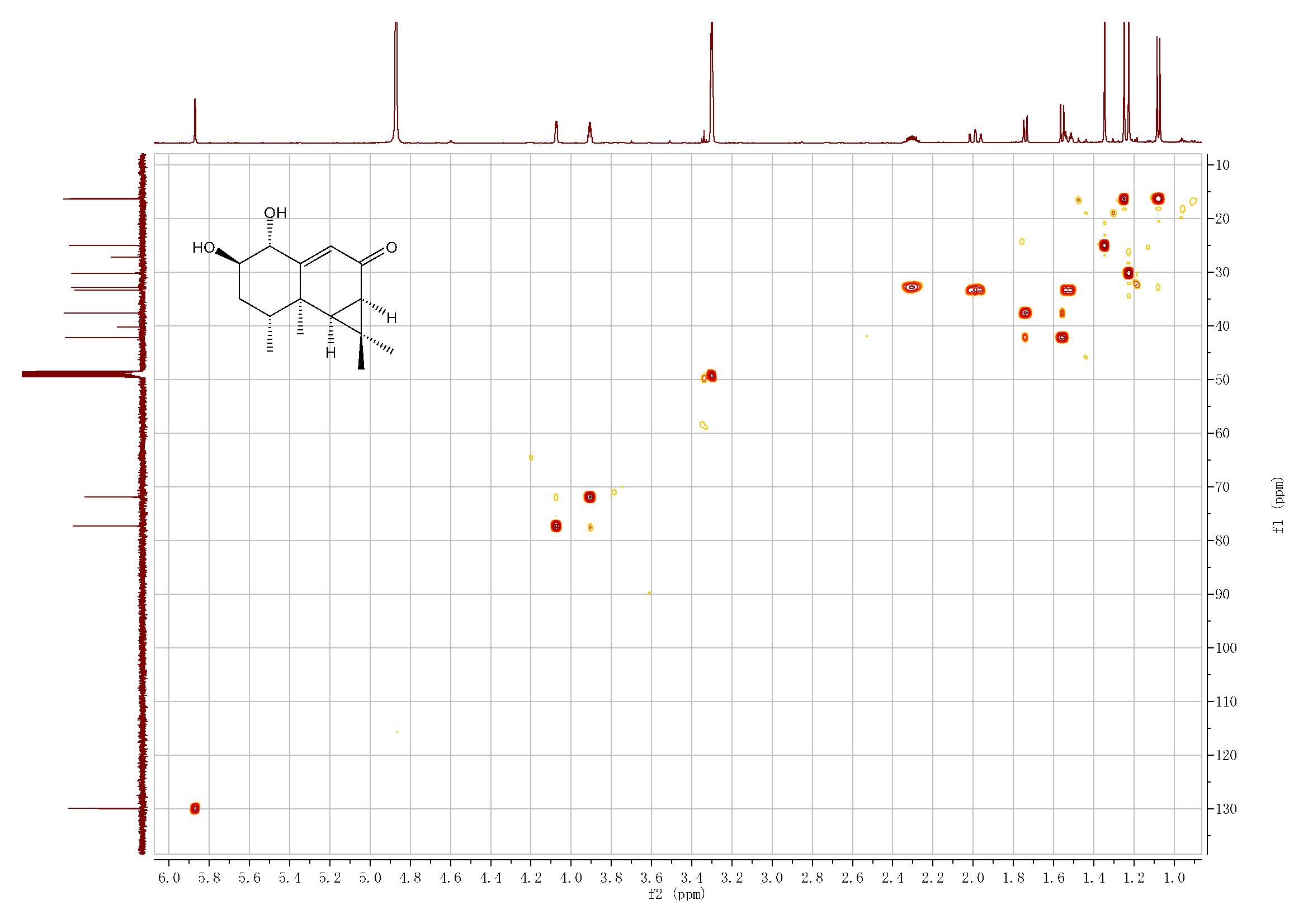


**S15.** ROESY spectrum (500 MHz, CD_3_OD) of 1*α*,2*β*-dihydroxyaristolone (**2**).


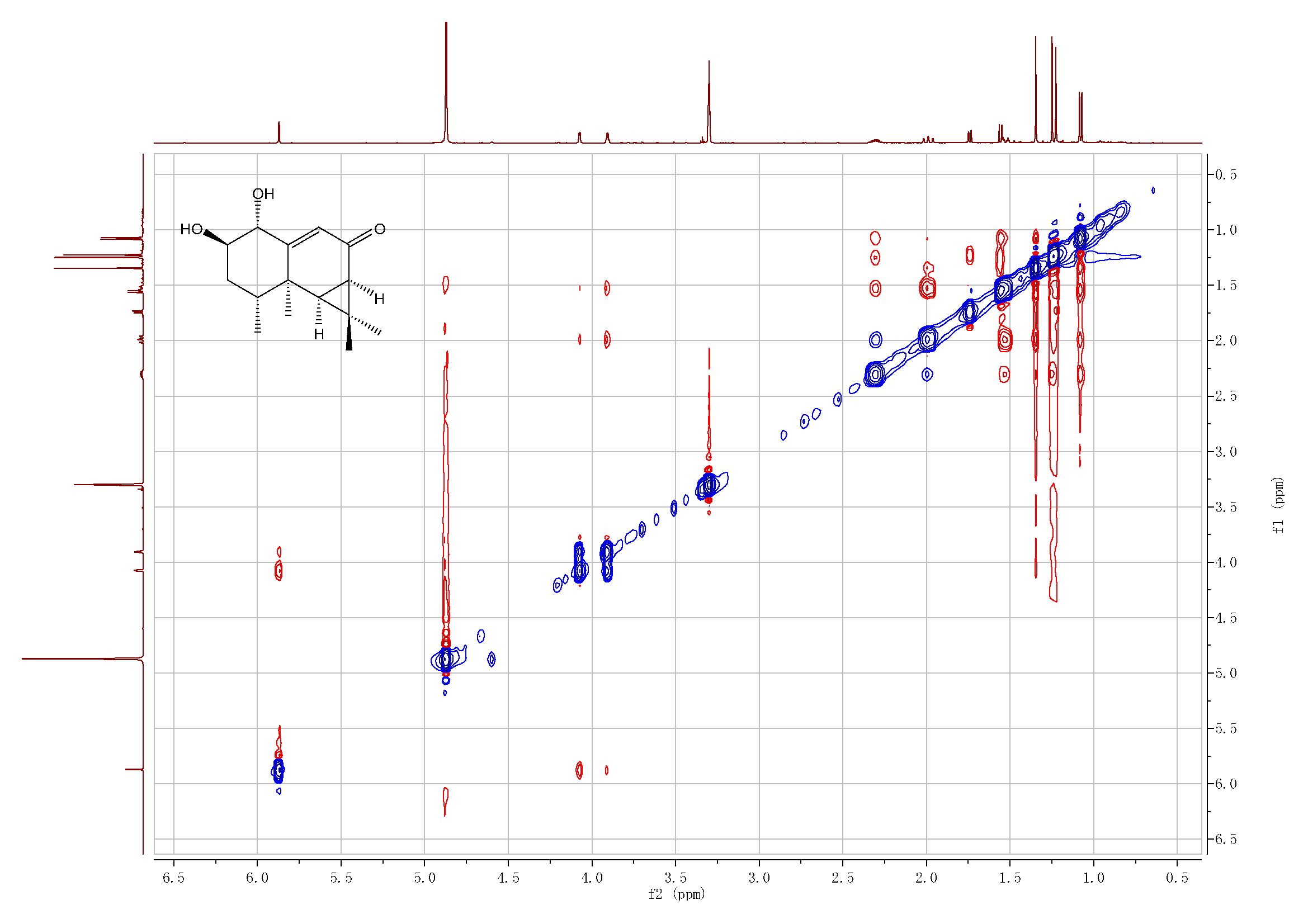


**S16.** ^1^H NMR spectrum (500 MHz, CDCl_3_) of 9-epidebilon (**3**).


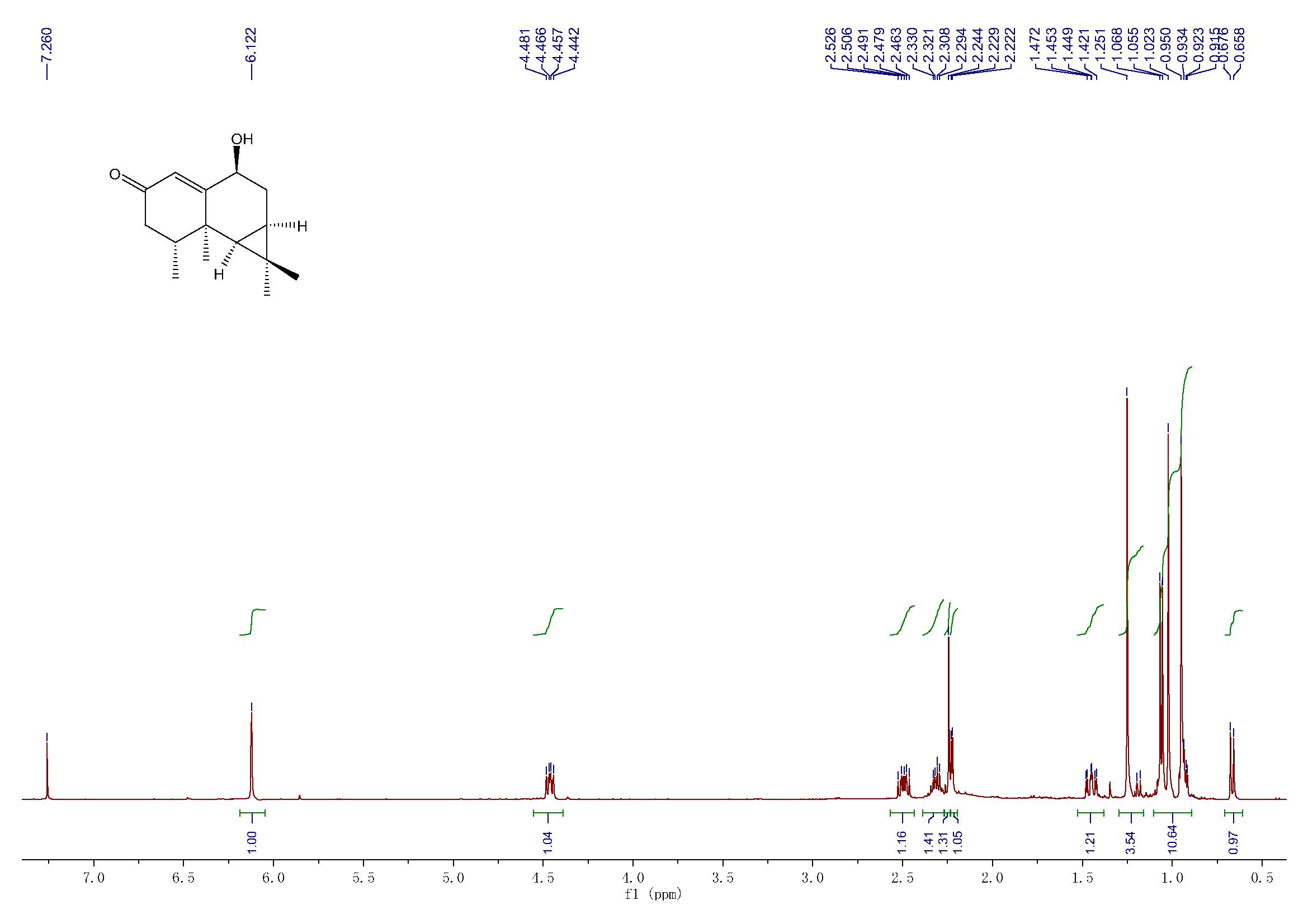


**S17.** ^13^C NMR spectrum (125 MHz, CDCl_3_) of 9-epidebilon (**3**).


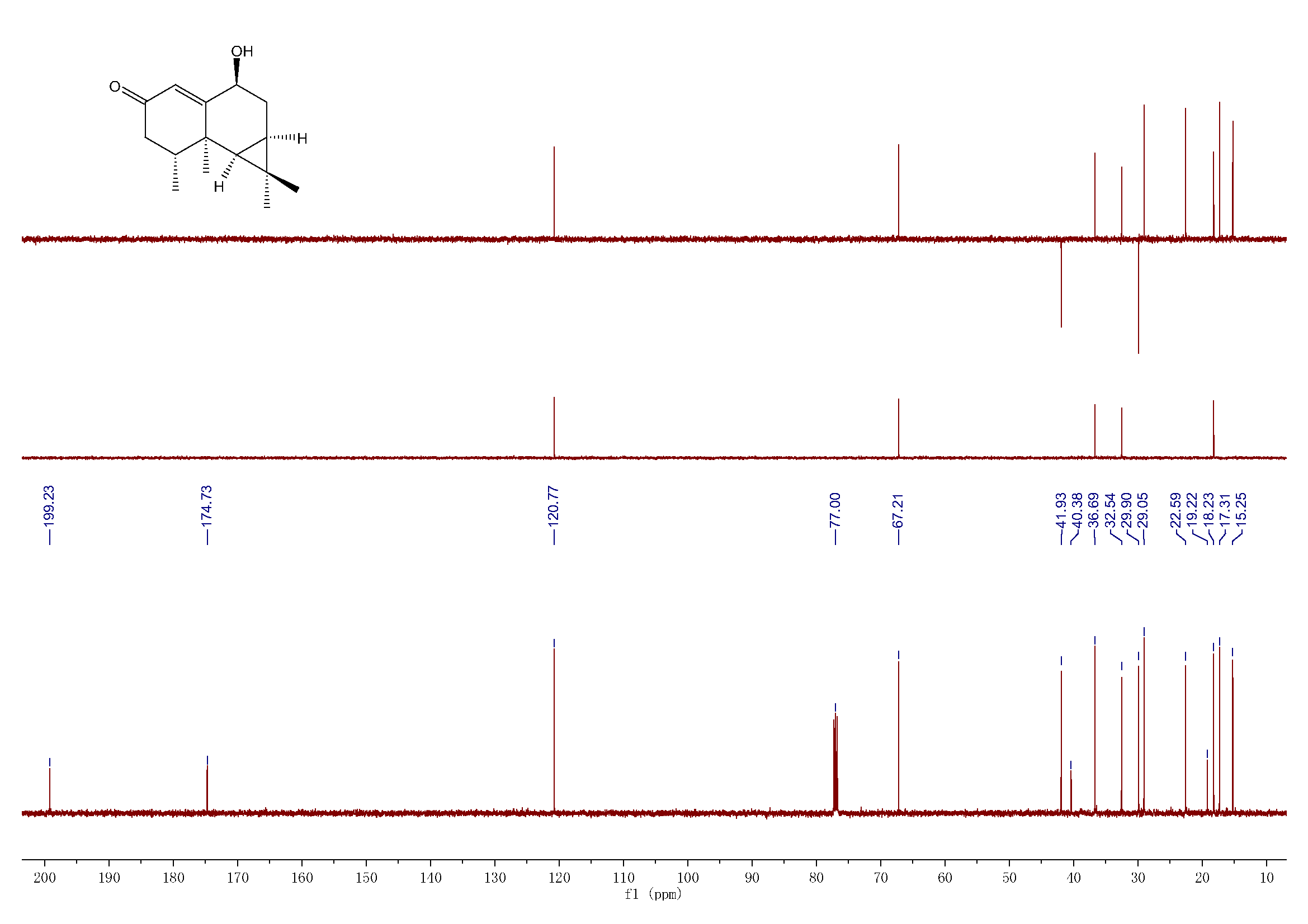


**S18.** HMBC spectrum (500 MHz, CDCl_3_) of 9-epidebilon (**3**).


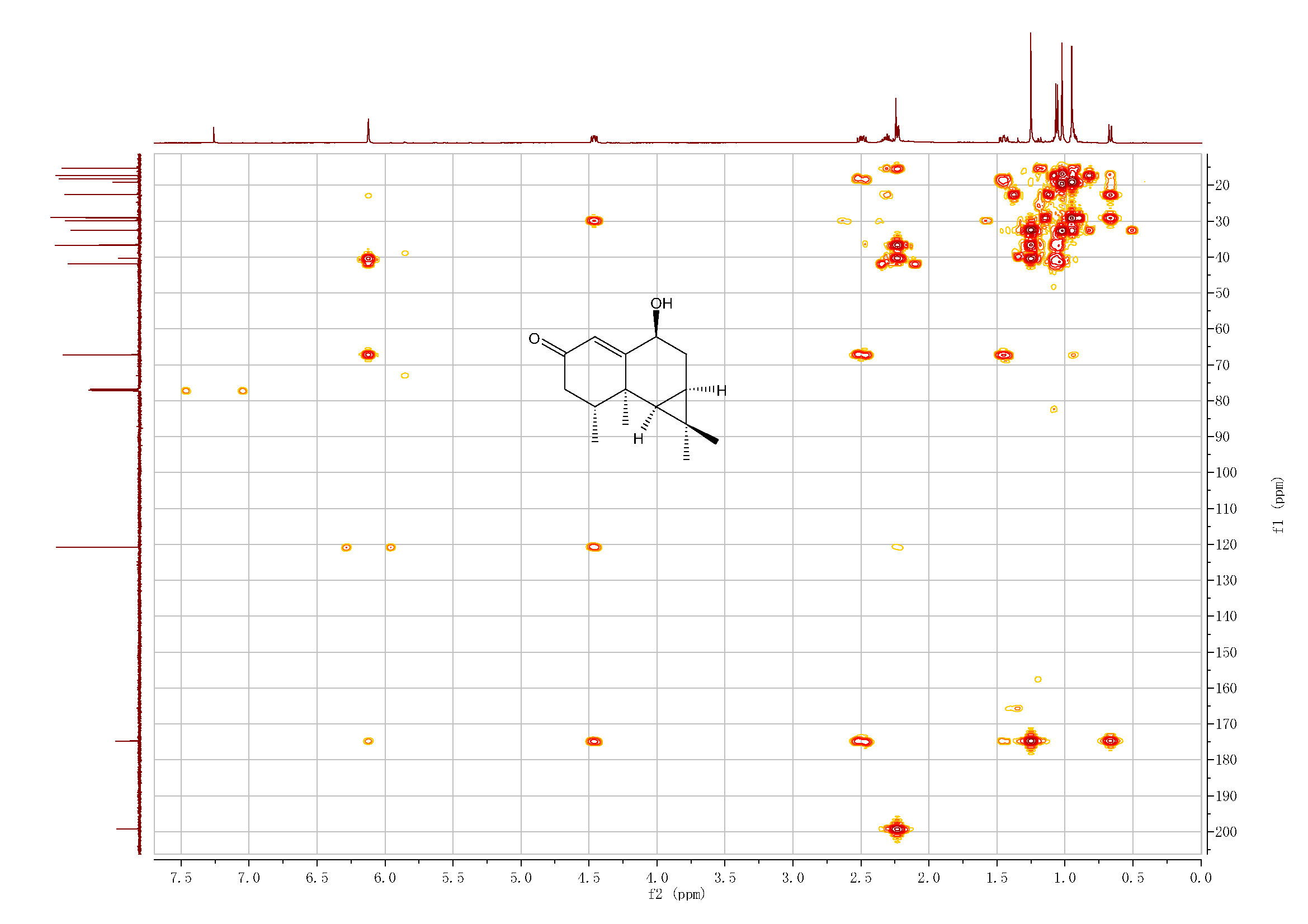


**S19.** HSQC spectrum (500 MHz, CDCl_3_) of 9-epidebilon (**3**).


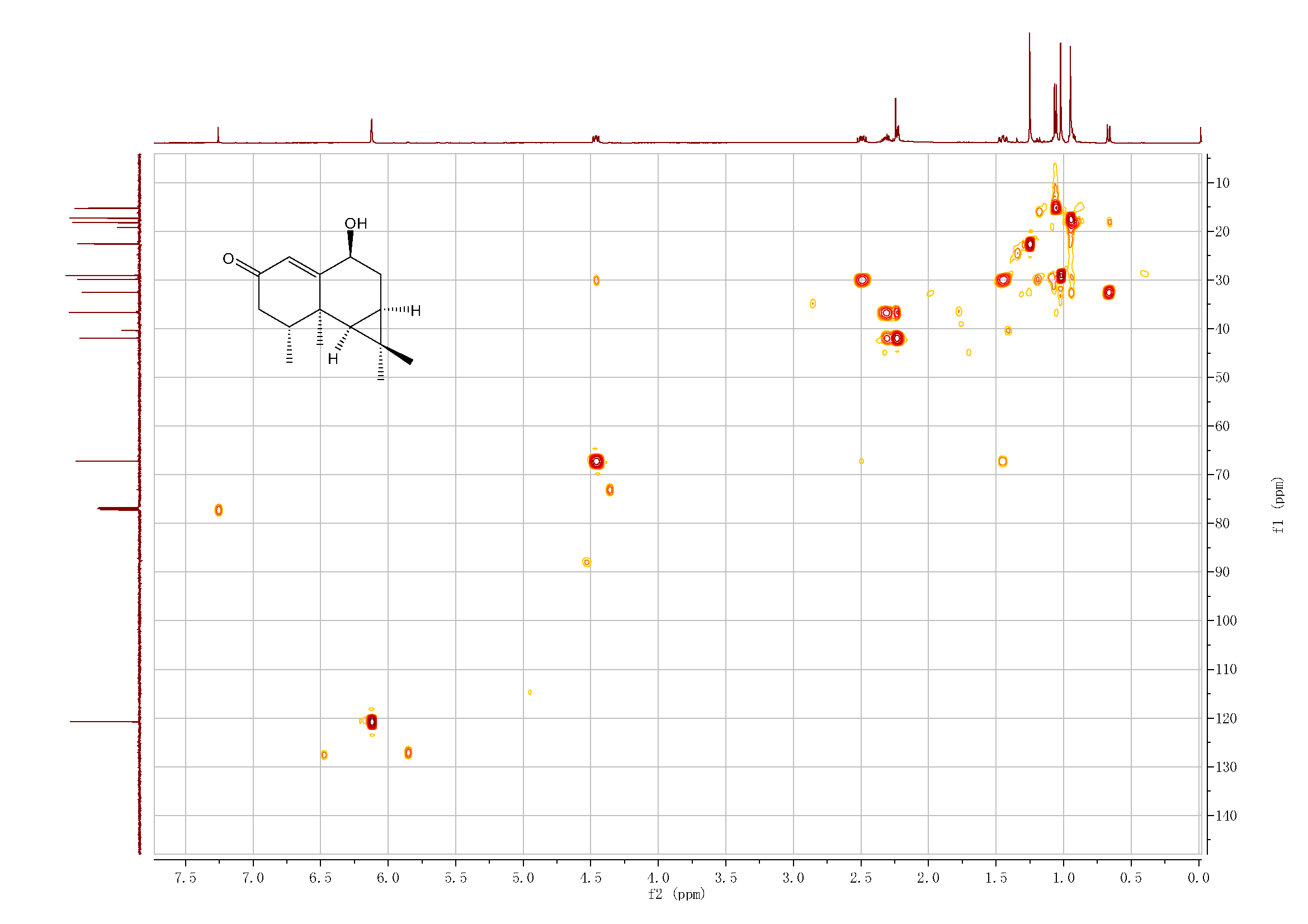


**S20.** ROESY spectrum (500 MHz, CDCl_3_) of 9-epidebilon (**3**).


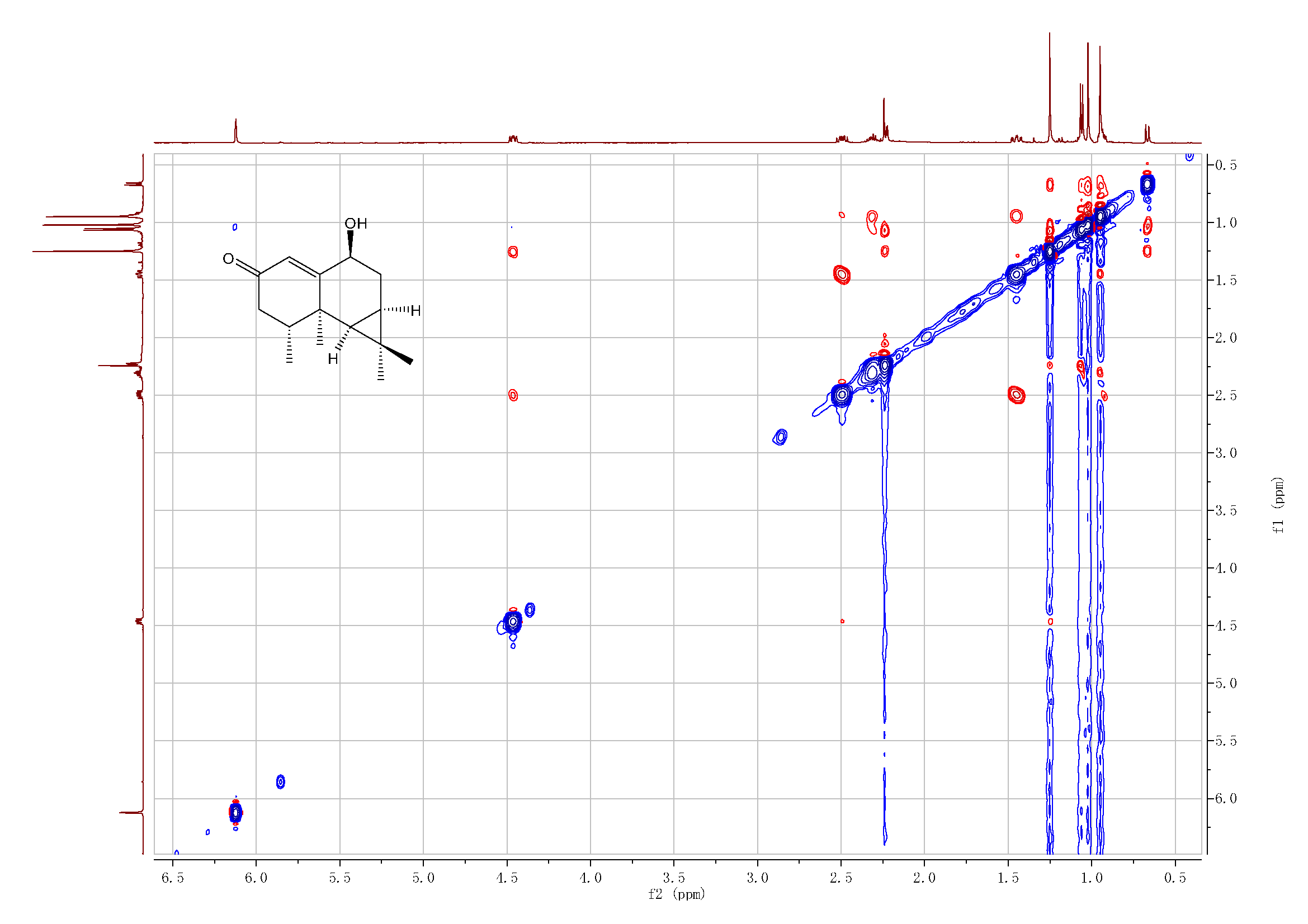


**S21.** ^1^H NMR spectrum (500 MHz, CDCl_3_) of 3′-hydroxynardoaristolone A **(4)**.


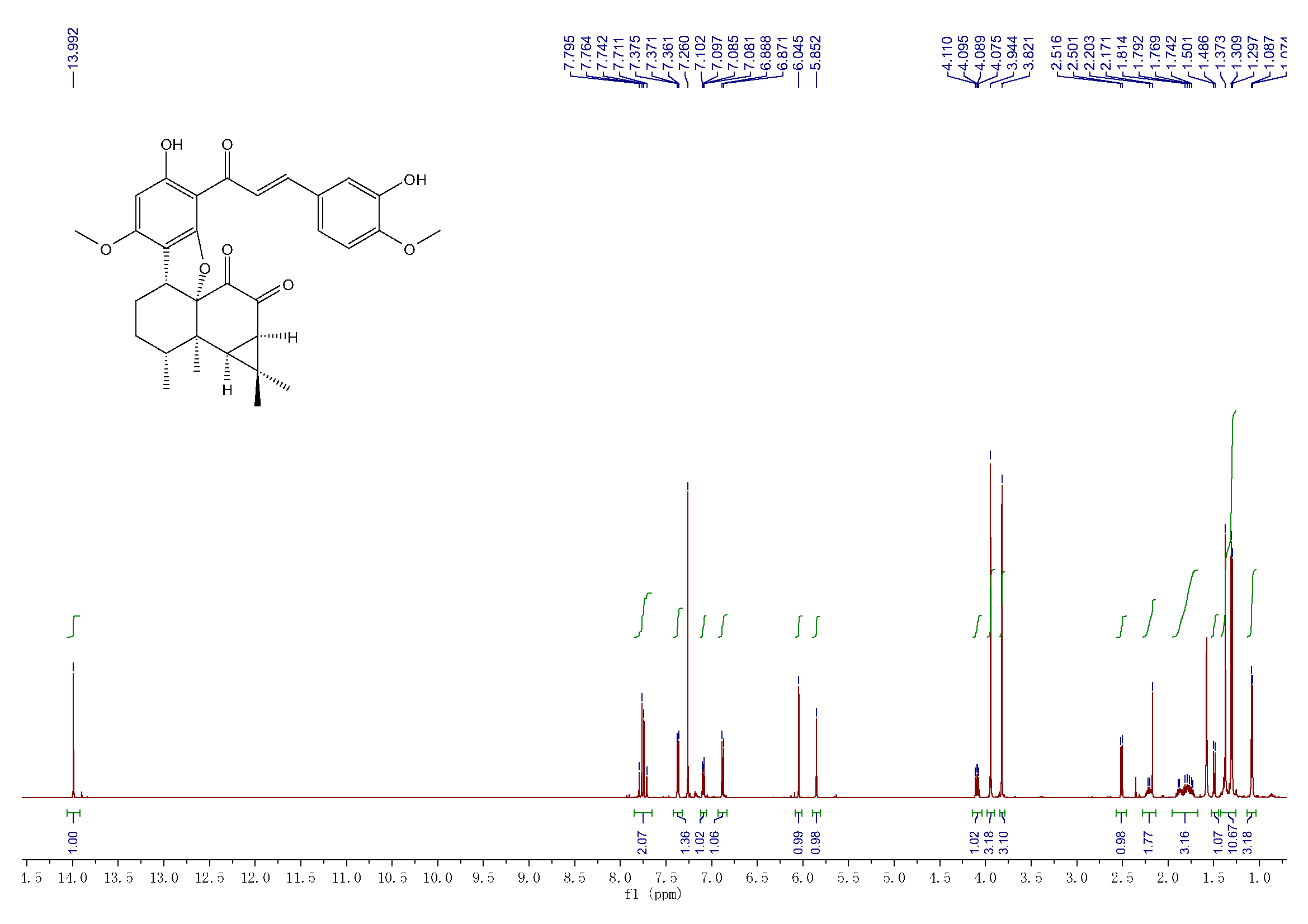


**S22.** ^13^C NMR spectrum (125 MHz, CDCl_3_) of 3′-hydroxynardoaristolone A **(4)**.


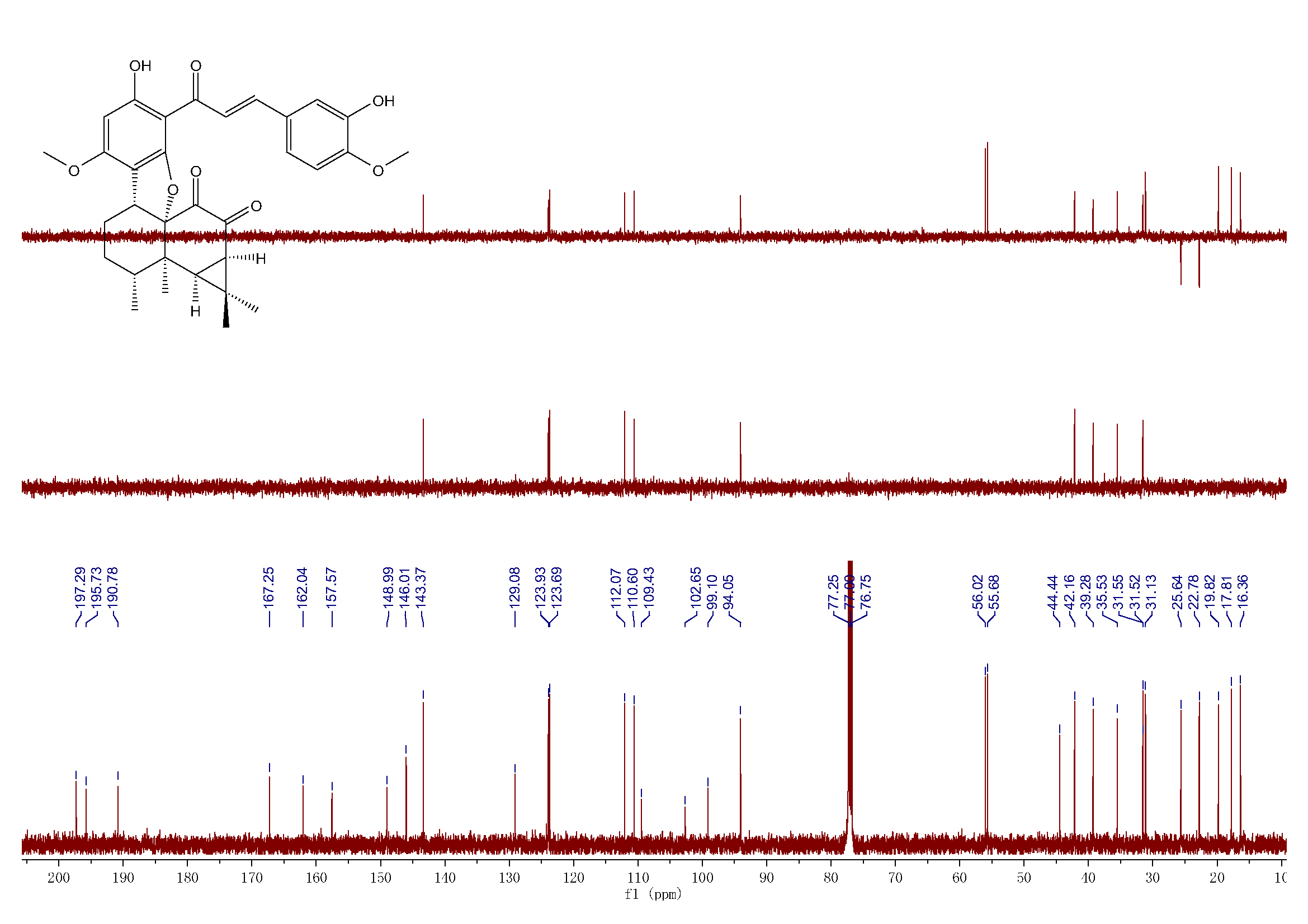


**S23.** HMBC spectrum (500 MHz, CDCl_3_) of 3′-hydroxynardoaristolone A **(4)**.


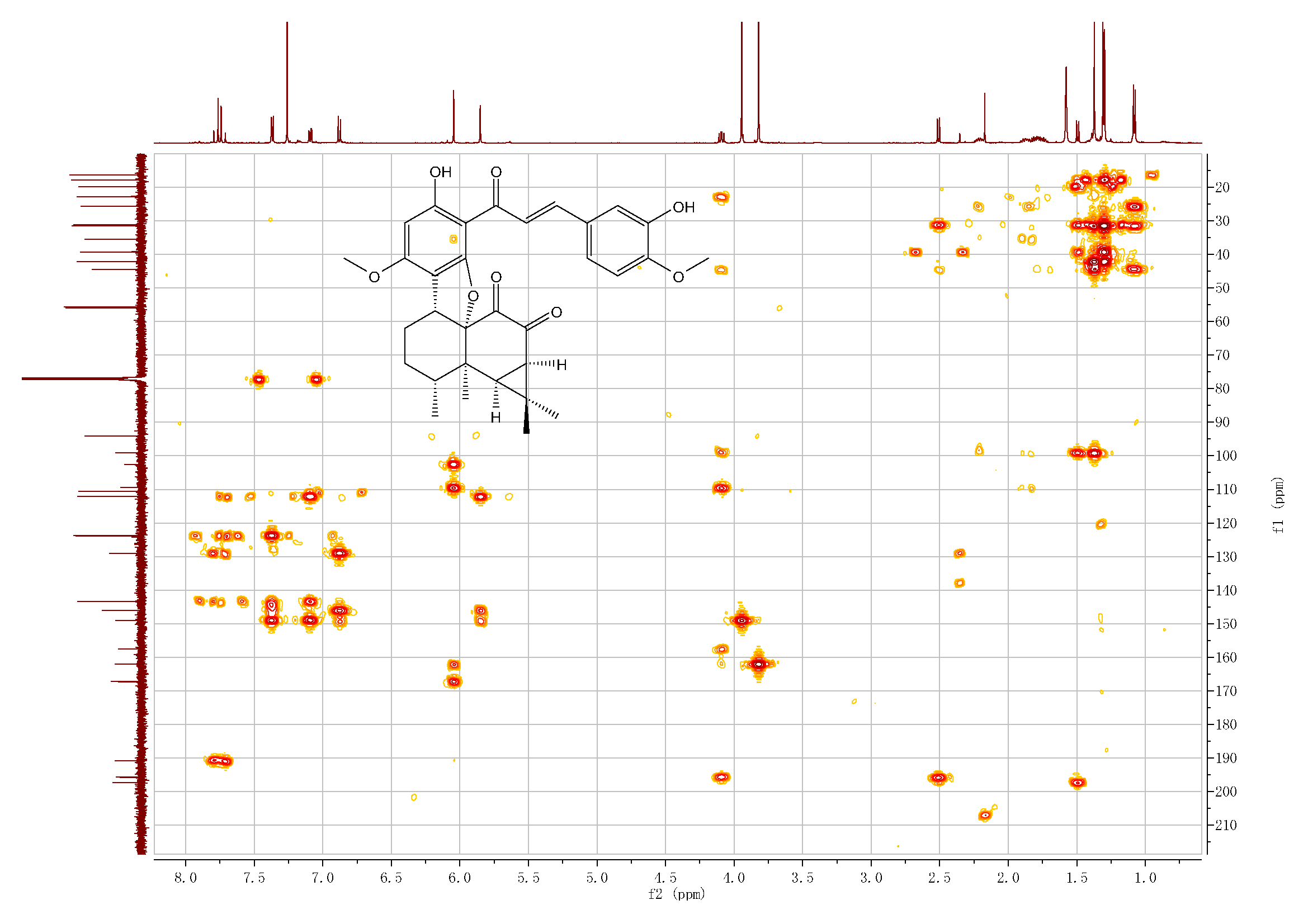


**S24.** HSQC spectrum (500 MHz, CDCl_3_) of 3′-hydroxynardoaristolone A **(4)**.


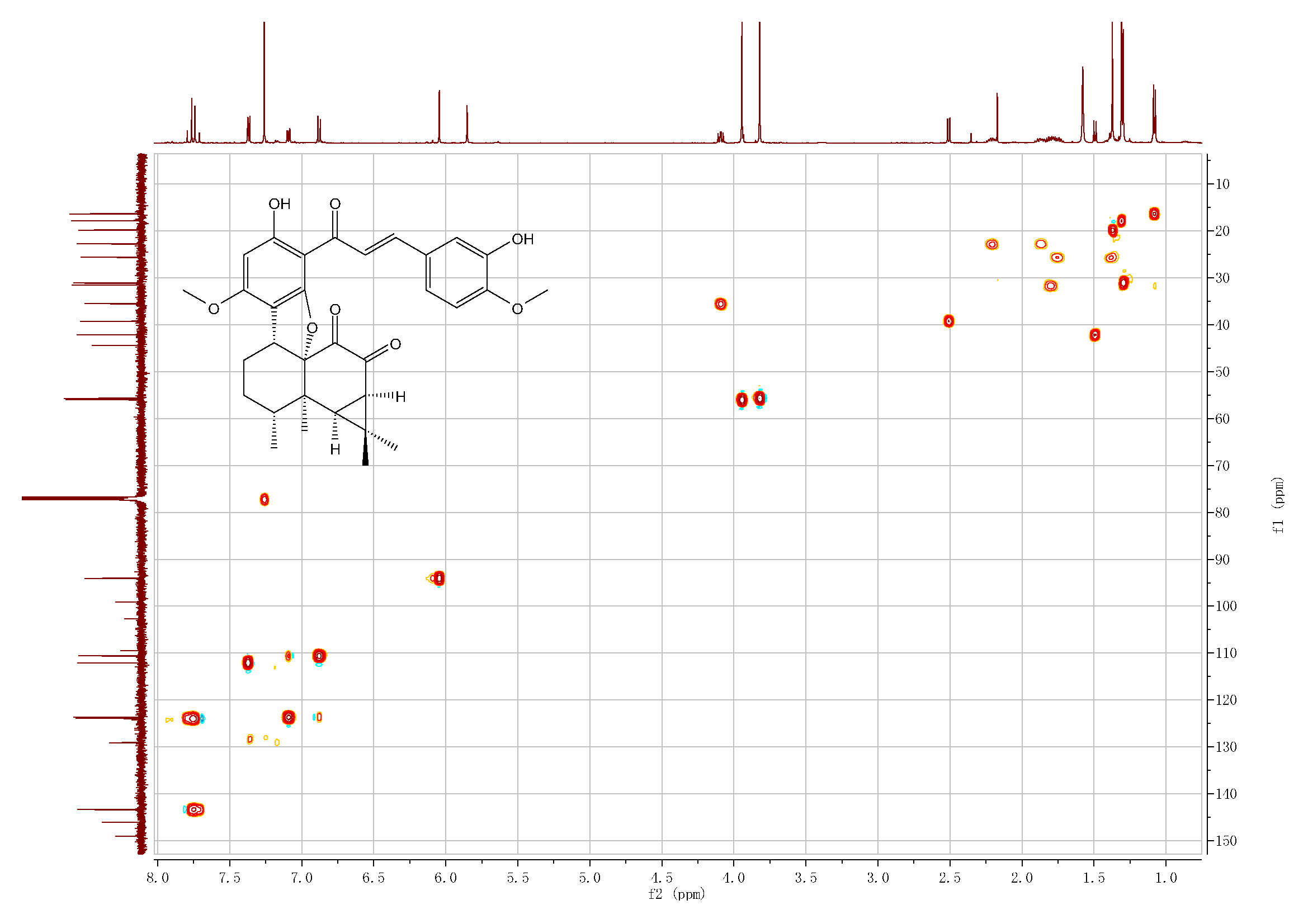


**S25.** ROESY spectrum (500 MHz, CDCl_3_) of 3′-hydroxynardoaristolone A **(4)**.


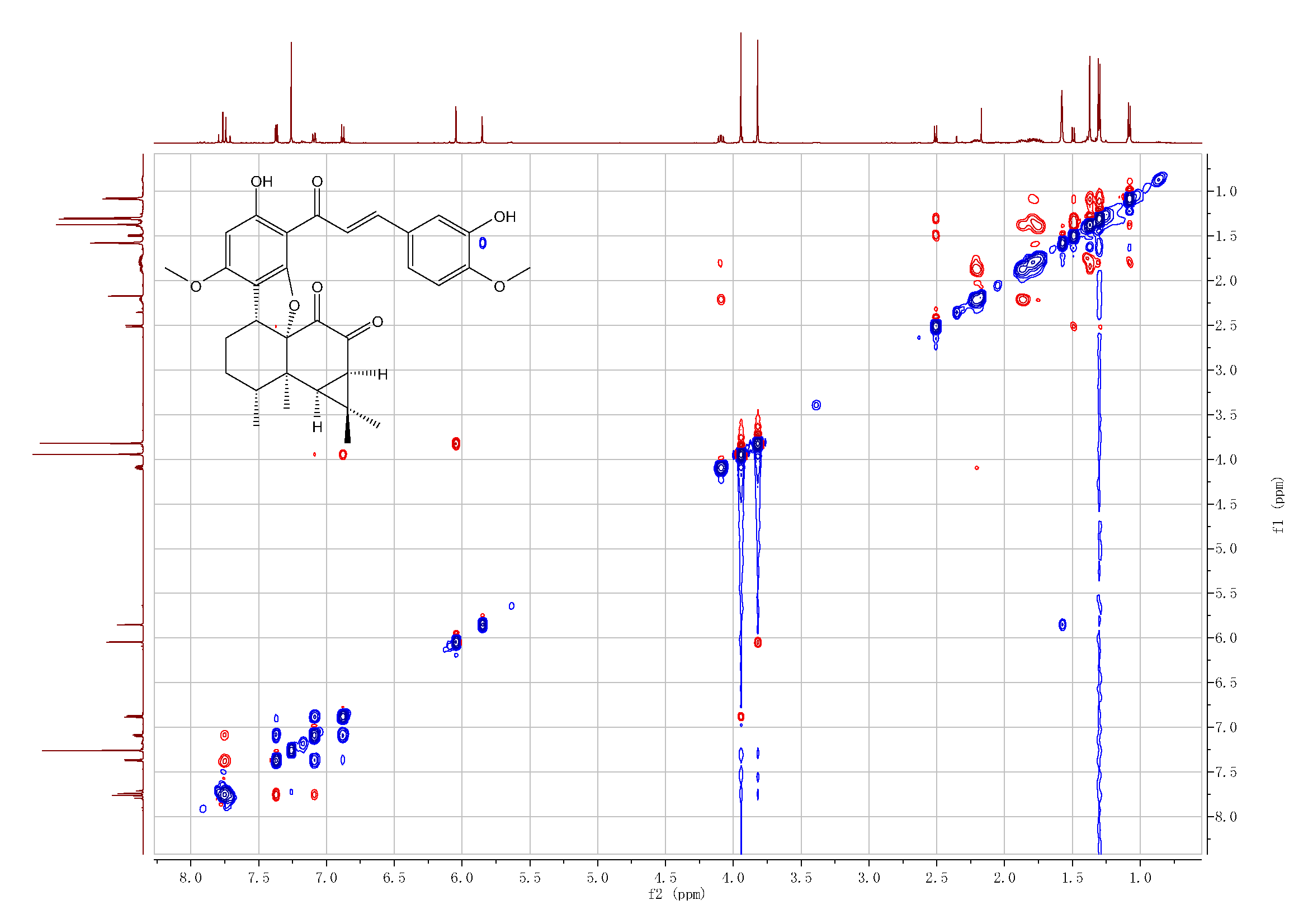

Supplement: Supplementary file 1 — Supplementary material 1 (DOC 1350 kb) [file 13659_2019_200_MOESM1_ESM.doc]
